# A 3D Model of the Effect of Tortuosity and Constrictivity on the Diffusion in Mineralized Collagen Fibril

**DOI:** 10.1038/s41598-019-39297-w

**Published:** 2019-02-25

**Authors:** Fabiano Bini, Andrada Pica, Andrea Marinozzi, Franco Marinozzi

**Affiliations:** 1 Department of Mechanical and Aerospace Engineering, “Sapienza” University of Rome, via Eudossiana, 18-00184 Rome, Italy; 2 Orthopedy and Traumatology Area, “Campus Bio-Medico” University, via Alvaro del Portillo, 200-00128 Rome, Italy

## Abstract

Bone tissue is a hierarchically structured material composed at the nanoscale by an organic matrix of collagen type I, apatite mineral and water. We considered an idealized 3D geometrical model of the mineralized collagen fibril in order to analyze the influence of structural factors, i.e. tortuosity, constrictivity, on the water effective diffusivity. The average values of the factors investigated in the diffusivity are computed on 5000 iterations by means of the Montecarlo method. The input parameters of the numerical model are the geometrical dimensions of the apatite mineral, collagen fibrils and their spatial orientation obtained with random extractions from Gaussian probability distribution functions. We analyzed the diffusion phenomenon for concentration gradients parallel to three orthogonal directions (Length, Width and Thickness) and for different scenarios, namely low, intermediate and high apatite volume fraction. For each degree of volume fraction, in the thickness direction, the tortuosity assumes greater values, up to two orders of magnitude, in comparison with the tortuous factors computed in the other directions, highlighting the anisotropy of the nanostructure. Furthermore, it was found that the tortuosity is the dominant parameter which control the effective transport properties within the mineralized collagen fibrils.

## Introduction

Diffusion phenomena in porous media have been studied extensively with the aim of a thorough understanding of the associated transport mechanism and the impact on the structure properties. The application of porous media theories to biological tissues has led to significant advances in the analysis of the structural characteristics of the medium^1–3^ and in the investigation of physiological processes, e.g. intercellular signaling^4^, diffusion of nutrients. With a particular focus on bone tissue, transport phenomena assume an important role also in the mechanosensing system which governs bone remodeling. A detailed understanding of the structure and properties of this complex porous system and their influence on the mass transport mechanism could give a contribution on crucial issues including alterations of bone tissue due to degenerative pathologies, e.g. osteoporosis^5^, osteoarthritis^6,7^.

Bone is a hierarchical composite biomaterial^8,9^. At the nanoscale, it is characterized by an assembly of 300 nm long and 1.5 nm thick collagen molecules which provides the site for mineral nanoparticles and water diffusion^8^. Namely, the mineralized collagen fibril contains 60–70 wt% plate-shaped apatite crystals, 20–30 wt% type I collagen fibrils and 10–20 wt% water^9^. The percentages vary depending on bone site, age, health conditions and environmental factors, resulting in an extremely heterogeneous structure^8,10^.

Collagen arrangement can be described by the quarter stagger-overlap configuration proposed by Hodge and Petruska^11^, that leads to a pattern of gap and overlap regions in the longitudinal direction of the fibril. The mineral nucleates first in the collagen gap region and successively extends in the pore spaces in the overlap zones^9^. An essential feature is the co-alignment between the apatite crystals and the collagen long axis^12–15^. Namely, the crystallographic *c-axes* of the apatite platelets lie within ±15–20° of one another rather than being perfectly parallel^14,15^. Moreover, Atomic Force Microscope imaging^16^ showed that, in the longitudinal direction of the fibril, the apatite minerals replicate the periodicity of the collagen matrix.

The 3D organization of the mineralized collagen fibril and the amount of mineral have a fundamental role in the structural and physiological functions of bone tissue. In pathological conditions, e.g. osteoporosis, zones with pronounced variations of mineral volume fraction (VF) are encountered^1,2,9,10,13,14,17–19^, thus analysis of the nanostructure arrangement could result useful for the characterization of diseases. Backscattered electron imaging methods^20,21^ report a mineral content for the human bone matrix between 0% and 43%. It is well established in literature that the apatite length can vary in the range 40 to 170 nm^16,22^, its width from 10 to 80 nm^13^ and its thickness from 2 to 5 nm^13^, but few considerations are available with regard to the distance between adjacent mineral platelets and their spatial arrangement^15,23^. The present model considers a staggered arrangement including overlapping of the apatite crystals in the longitudinal direction, while in the thickness and width direction parallel layers are assumed, in agreement with the studies of Hodge and Petruska^11^ and Vercher-Martinez *et al*.^23^.

The sizes of the mineral and the distances between the platelets are set in order to analyse three different mineralization percentage: low (7 vol%)^23^ and high (42 vol%) calculated in 3D, according to Vercher-Martinez *et al*.^23^, equivalent to the 52% vol. related to a 2D model adopted from Nikolov *et al*.^24^. Similarly, an intermediate value in 3D was calculated (32 vol%, see Supplementary Figs S1–S3) equivalent to the 42% vol. for a 2D model adopted from Jäger *et al*.^25^.

The third main component of bone nanostructure is water and it may be distinguished into two main types according to its free or bound state. In this study, we analyse free bone water at the collagen-apatite level of porosity (about 10 nm)^26^ that contributes to important processes in mineralization, bone growth and healing through the transmission of remodelling signals^3^ or plays a structuring role by orienting apatite crystals^27^.

The impact of the orientation and arrangement of the bone structure on the transport properties have been already investigated in several experimental^18^ and computational studies^28^. Experiments performed by Marinozzi and co-workers^18^ allowed to highlight a marked anisotropy of the arrangement of the apatite crystals along the three main axes of the trabecula. In fact, from an analysis of the experimental data by means of a genetic algorithm^18^, three different values of the diffusion coefficient along the main axes of a human trabecula (Length L, Width W and Thickness T) were achieved. As expected, the diffusivity values, i.e. [D_L_ = 1.03·10^−9^, D_W_ = 1.26·10^−10^, D_T_ = 1.16·10^−11^ (m^2^·s^−1^)] along the axes of the trabecula results minor than the diffusion coefficient of water in a homogenous medium, i.e. D_0_ = 2.66·10^−9^ (m^2^·s^−1^)^29^. The observed anisotropic behaviour was confirmed also by molecular dynamics studies performed by Di Tommaso and colleagues^30^.

In this study, we improve a previous analytical model^28^ of the diffusion phenomenon at the collagen-apatite level of porosity, i.e. 10 nm^26^. The crucial goal of this work is a detailed investigation of the structural factors which control the effective diffusion coefficient within the bone nanostructure. The structure of the collagen fibril reinforced with mineral particles leads to a deviation of the diffusion paths of water from straight lines due to the presence of solid components. Thus, transport mechanism is influenced by geometric features and structural configuration, which cause a decrement of the effective diffusion coefficient with respect to the diffusivity in a homogeneous medium^31^.

Predictions of the effective diffusivity achieved analytically in function of the structural nature of porous media were developed first in the fields of chemical engineering and geological sciences^32,33^. Subsequently, these models have been extended to the brain tissue^34^, and more recently, to the mineralized collagen fibrils^28^. The effect of the spatial configuration on the transport properties may be analyzed by means of three factors: porosity of the material, tortuosity of the flow path, which characterizes the sinuousness of the solutes streamlines through the heterogeneous medium and constrictivity, which represents the so-called bottleneck effect^33,35^.

The tortuous factor (τ) is a quantitative measure of the reduction of diffusive flux caused by the sinuous path imposed by the obstacles compared to the straightest path in an unrestricted medium, in the direction of the flow^36^. The complex nature of porous medium creates difficulties in order to quantify the tortuosity. Different approaches for estimating this geometric factor have been developed: diffusion experiments^18^, ultrasonic reflectivity methods^37^, NMR measurements^38^, analytical models^36,39,40^ or 3D image analysis^41^.

The constrictivity (δ) is a dimensionless factor and it assesses the impact of the variation of pores cross section on the mass transport^33^. It becomes important only if the size of the solute is comparable to the dimensions of the pores^42^. Recently, new methods have been proposed in order to determine the constrictivity factor directly from disordered porous medium from tomography and 3D image analysis^36^ or by means of experimental methods as mercury intrusion porosimetry^43^. Nevertheless, to the best of our knowledge, image analysis on bone tissue which could provide a quantification of the topological features, i.e. tortuosity and the constrictivity, directly from tomographic 3D reconstruction is still absent.

In this work, we implemented a numerical model that predicts the effective diffusivity based on an idealized geometry of the mineralized collagen fibrils and analyses the influence of the tortuosity and constrictivity on the mass transport properties. Although electron microscopic tomography has been utilized to study the 3D spatial arrangement of mineral crystals within the fibrils, the resolution of this technique, 4–6 nm, is not sufficient to resolve the thickness of mineral platelets embedded in the densely packed collagen fibrils^14,15^. Therefore, there are very few available data about the packing of mineral crystals in the radial direction of the collagen fibrils.

Theoretical models of bone at the nanoscale can give remarkable insight on the tissue behaviour and its physical properties. Numerous approaches are presented in literature, spanning from continuum levels studies^25,28,44,45^ to atomic level simulations^3^.

In this work, the assessment of the effective diffusion coefficient of water has been conducted considering a continuum mechanics approach. We reconstructed the bone nanostructure as a periodically staggered model and geometrical expressions were derived for the tortuosity of solute streamlines and the constrictivity, as functions of the model parameters. Numerical values of the effective diffusion coefficients were achieved by means of the Monte Carlo method. Moreover, we analysed the impact of these structure parameters, i.e. tortuosity and constrictivity, on the reduction of the water diffusion coefficient and illustrated their dependence on the nanostructure VF.

## Results

**Figure 1:**
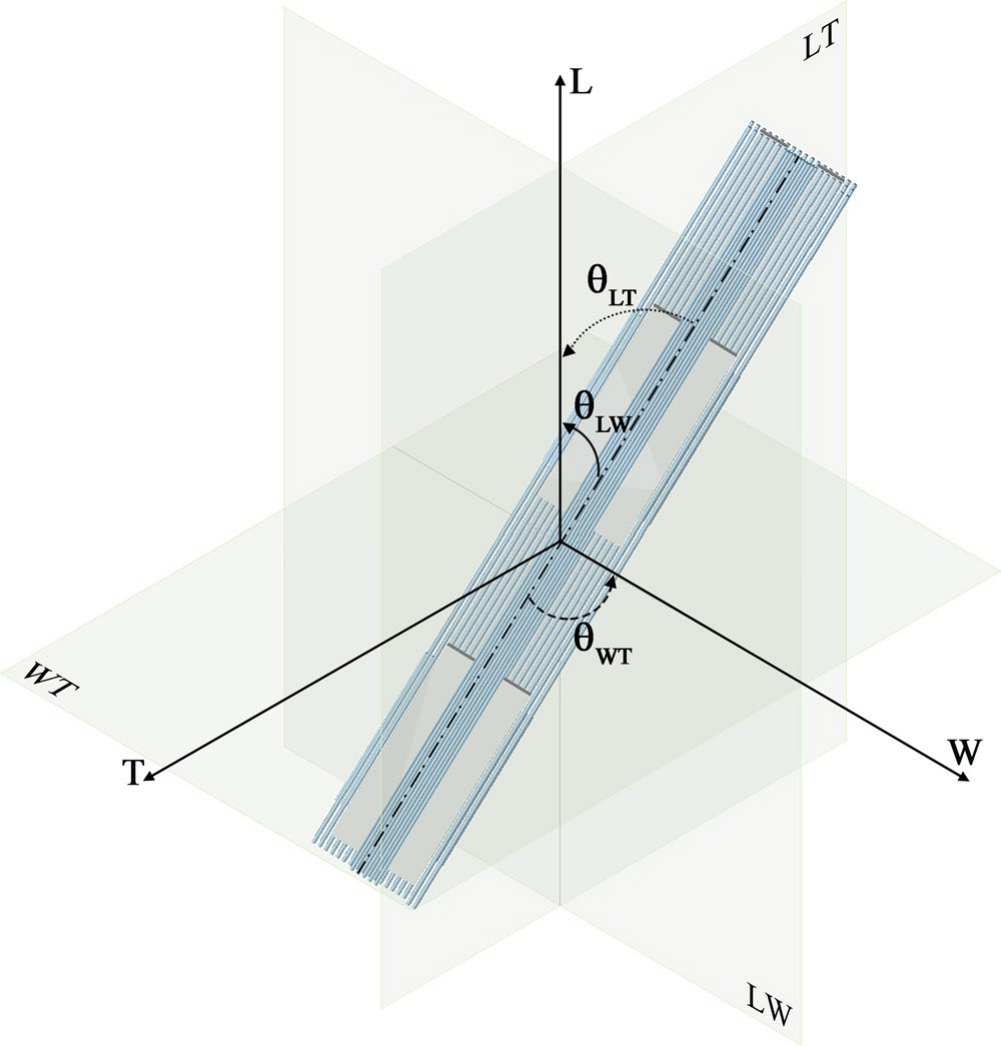
Schematic representation of the mineralized collagen fibril assumed as a composite of collagen fibrils (light blue region) and apatite platelets (grey region). We represent four unit cells of bone nanostructure in order to highlight the spatial arrangement of the mineral embedded in the collagen fibrils. Each unit cell is composed by three apatite platelets disposed in a staggered configuration along the longitudinal axis. We consider that the inclination of mineral with respect to the longitudinal axis L is identified by the angle θ_LT_ in the LT plane and respectively by θ_LW_ in the LW plane. In the WT plane, the inclination, θ_WT_, is considered with respect to the width axis W.

We analysed the diffusion phenomenon within a repeating unit cell of bone nanostructure composed by three platelets of apatite embedded in the collagen matrix (Fig. 1). We determined the diffusion coefficient of water assuming a concentration gradient parallel to the three axes, i.e. L, W, T, of a global coordinate system (CS_T_) fixed to the main axes of the trabecula, compliance with the system of reference used in the previous computational study^28^.

Experimental observations from TEM and SAXS studies^46,47^ highlight that the apatite minerals are locally parallel between them. Therefore, we introduce the assumption that the unit cell is composed of parallel platelets, while their orientation with respect to the axes of the CS_T_ is described by Gaussian probability density functions (PDFs) with mean 0 degrees and variable standard deviation. We considered 5000 realizations for each Gaussian PDF describing the unit cell inclinations. Overall, we investigated 5000 different Gaussian PDFs and for each one we computed the average of the 5000 realizations. We analysed different values of the degree of apatite volume fraction (V_f_A_), respectively 0.07, 0.32 and 0.42.

**Figure 2:**
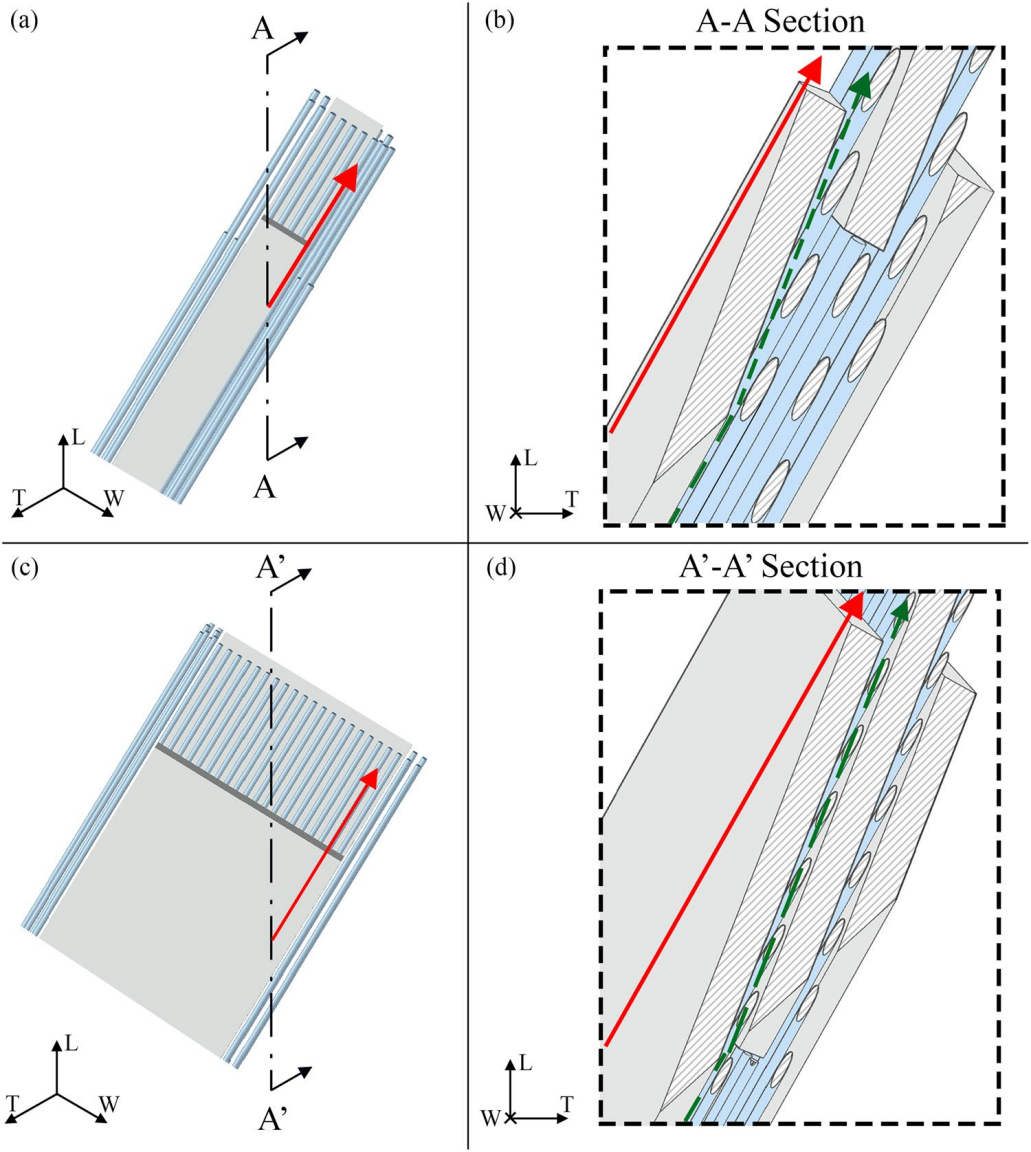
Graphical definition of the tortuosity for a flow parallel to the longitudinal direction (L). The apatite platelets configuration is characterized by an inclination of θ_LT_ = −20°; θ_LW_ = − 25°; θ_WT_ = 25° in both cases of V_f_A_ = 0.07 (**a,b**) and V_f_A_ = 0.42 (**c,d**). (**b**,**d**) Show sections of the model parallel to the LT plane. The green dashed lines illustrate the streamlines of the water molecule along the longitudinal direction. The red continuous lines represent the Euclidean distance between the path extremes. Sections of the two degrees of mineralization are in scale in order to highlight the changes in the geometry with the increase of the mineral VF.

**Figure 3:**
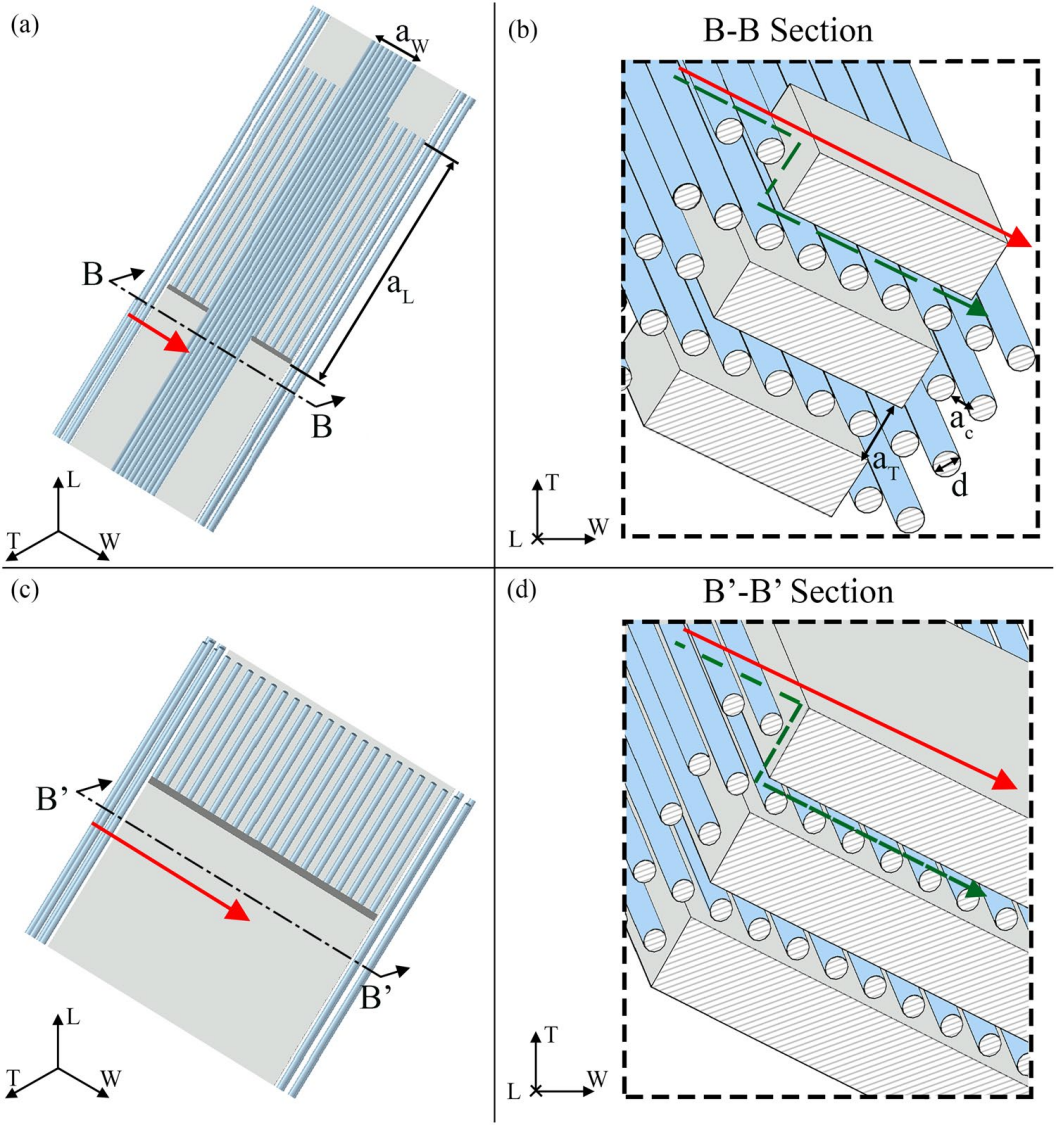
Graphical definition of the tortuosity for a flow parallel to the width (W) direction. The apatite platelets configuration is characterized by an inclination of θ_LT_ = −20°; θ_LW_ = −25°; θ_WT_ = 25° in both cases of V_f_A_ = 0.07 (**a,b**) and V_f_A_ = 0.42 (**c,d**). (**b,d**) Show sections of the model parallel to the WT plane. The green dashed lines illustrate the streamlines of the water molecule along the width direction. The red continuous lines represent the Euclidean distance between the path extremes. Sections of the two mineral content cases are in scale in order to highlight the changes in the geometry with the increase of the mineral VF. In (**a**,**b**) we also indicated the variables that characterize the distance between the platelets (a_L_, a_W_ and a_T_) and between the collagen fibrils (a_c_) of diameter (d).

**Figure 4:**
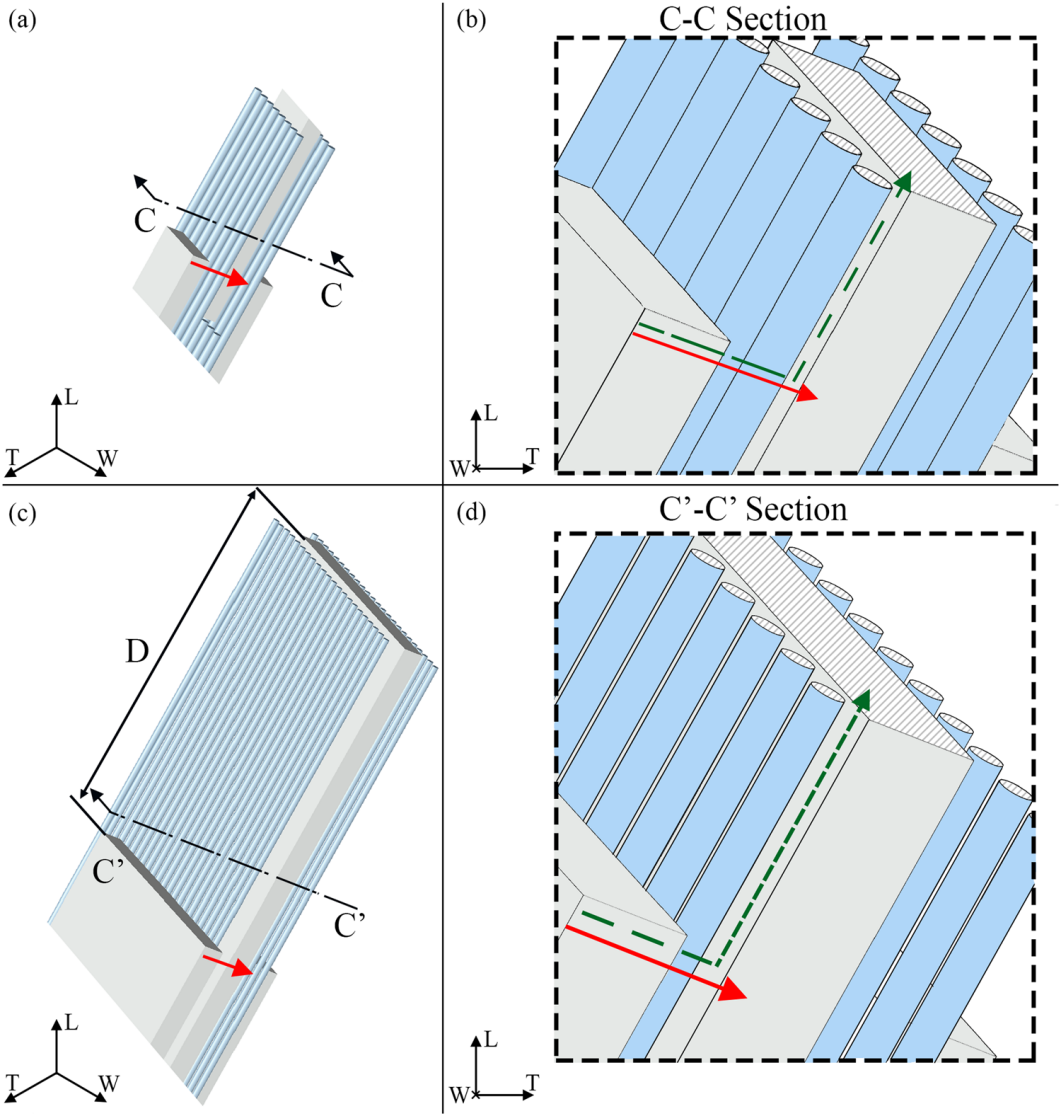
Graphical definition of the tortuosity for a flow parallel to the thickness (T) direction. The apatite platelets configuration is characterized by an inclination of θ_LT_ = −20°; θ_LW_ = −25°; θ_WT_ = 25° in both cases of V_f_A_ = 0.07 (**a,b**) and V_f_A_ = 0.42 (**c,d**). (**b,d**) Show sections of the model parallel to the WT plane. The green dashed lines illustrate the streamlines of the water molecule along the thickness direction. The red continuous lines represent the Euclidean distance between the path extremes. Sections of the two mineral content cases are in scale in order to highlight the changes in the geometry with the increase of the mineral VF. In (**c**) is showed the period length (D) between adjacent mineral platelets in the longitudinal direction.

**Figure 5:**
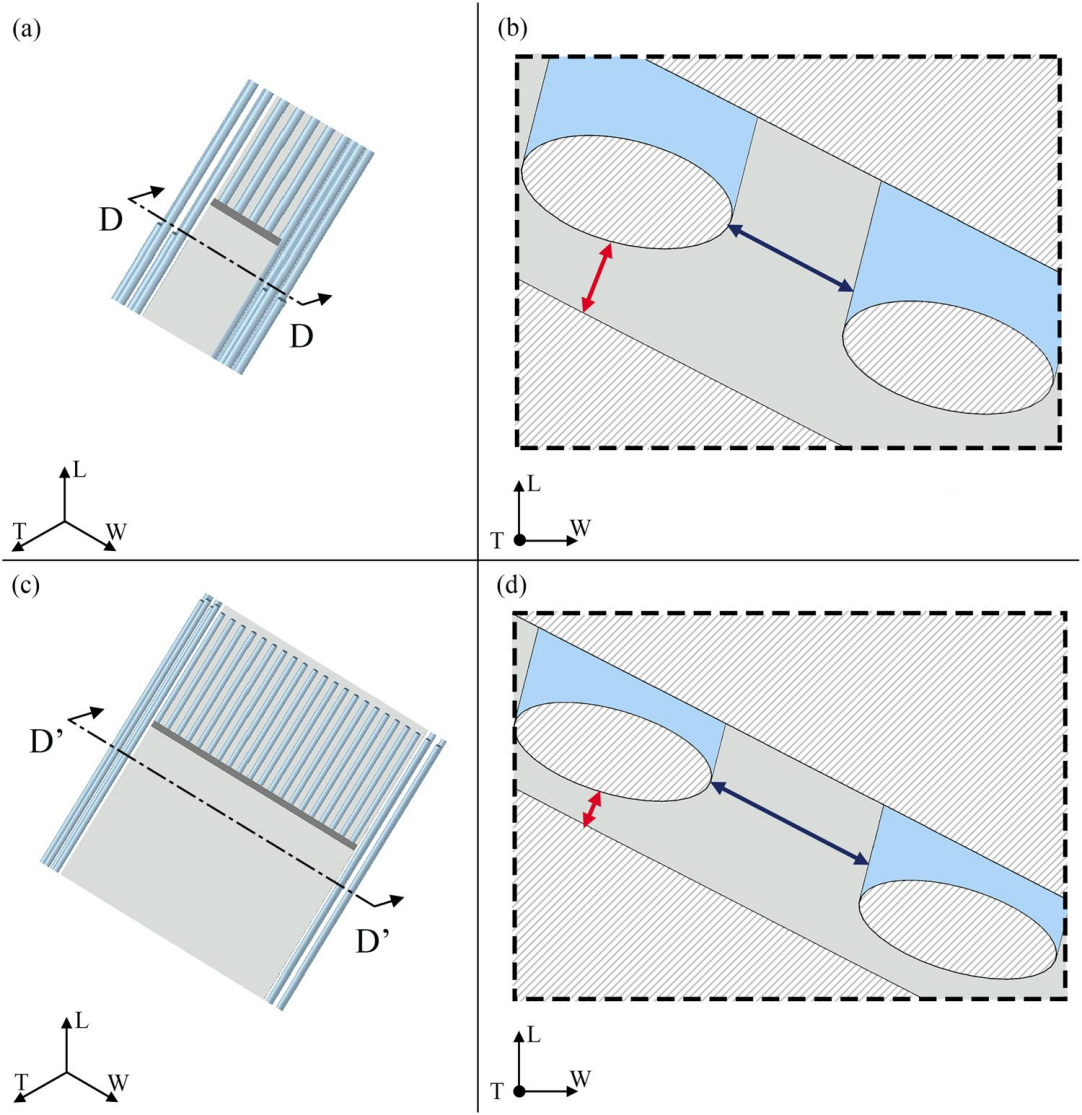
Graphical definition of constrictivity. The apatite platelets configuration is characterized by an inclination of θ_LT_ = −20°; θ_LW_ = −25°; θ_WT_ = 25° in both cases of V_f_A_ = 0.07 (**a,b**) and V_f_A_ = 0.42 (**c,d**). (**b,d**) Show sections of the model parallel to the WT plane. The dark blue and red double arrows represent the maximum and minimum cross sections respectively, considered for the calculation of the constrictivity factor in Eq. (6).

For each main plane, i.e. LT, WT and LW, we take into account the contributes to the transport resistance expressed by the tortuosity, i.e. the path length the molecule of water has to perform between two points with respect to a straight line in the direction of the flow (Figs 2–4), the constrictivity, i.e. the reduction of the flow due to the variations in the cross section of the passageway available for the water molecule path diffusion (Fig. 5) and the porosity.

**Figure 6:**
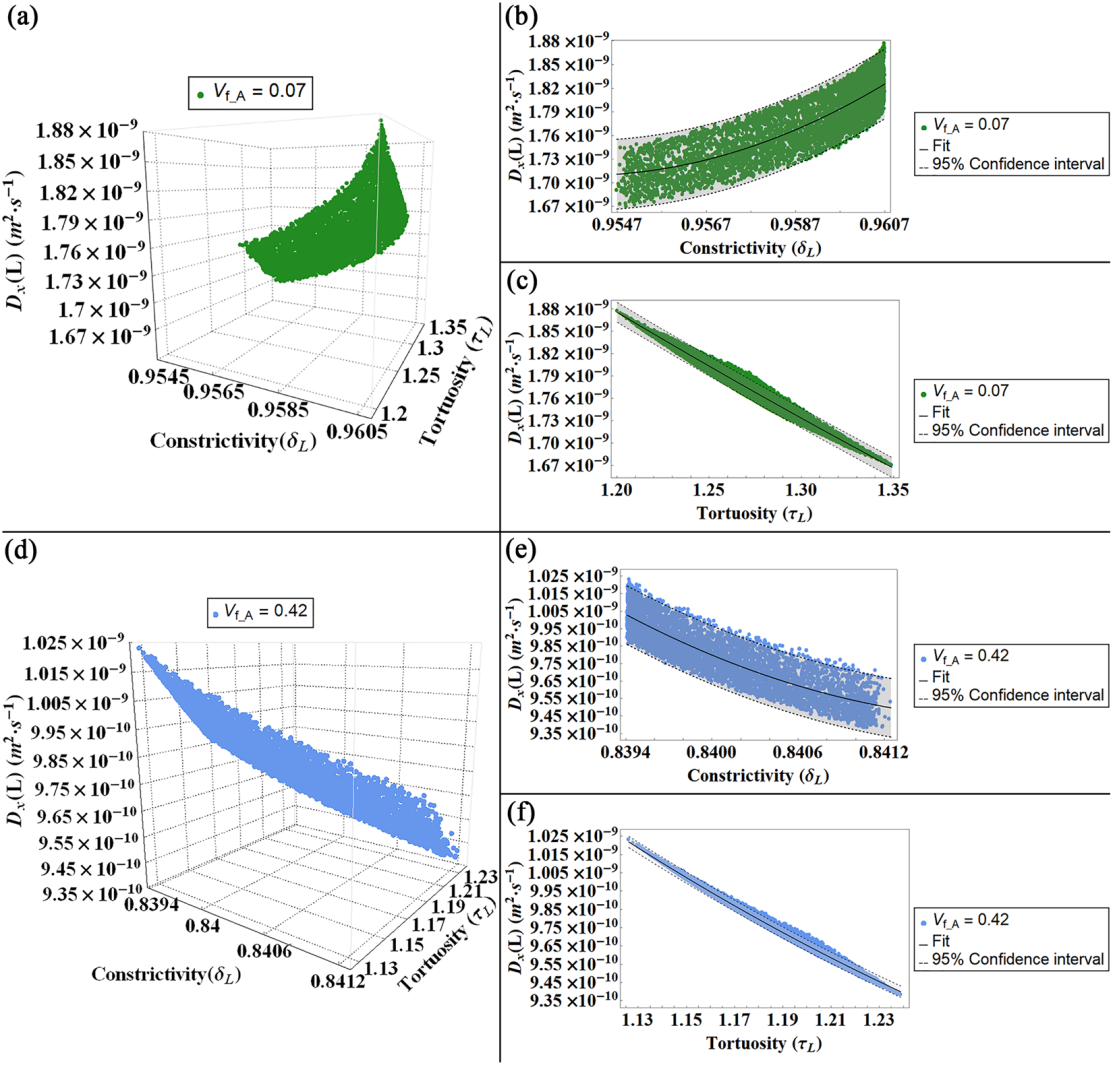
Diffusion coefficient (D_L_) for a flow parallel to the longitudinal axis (L) represented in function of the constrictivity and tortuosity factors for both models, i.e. V_f_A_ = 0.07 (**a–c**) and V_f_A_ = 0.42 (**d,f**): in (**a,d**) 3-D plot of the diffusivity D_L_ versus constrictivity and tortuosity. In (**b,e**) 2-D plot of D_L_ versus the constrictivity and in (**c,f**) 2-D plot of D_L_ versus the tortuosity. The colour bands in the 2-D plots indicates the Confidence Interval at 95 percent.

**Figure 7:**
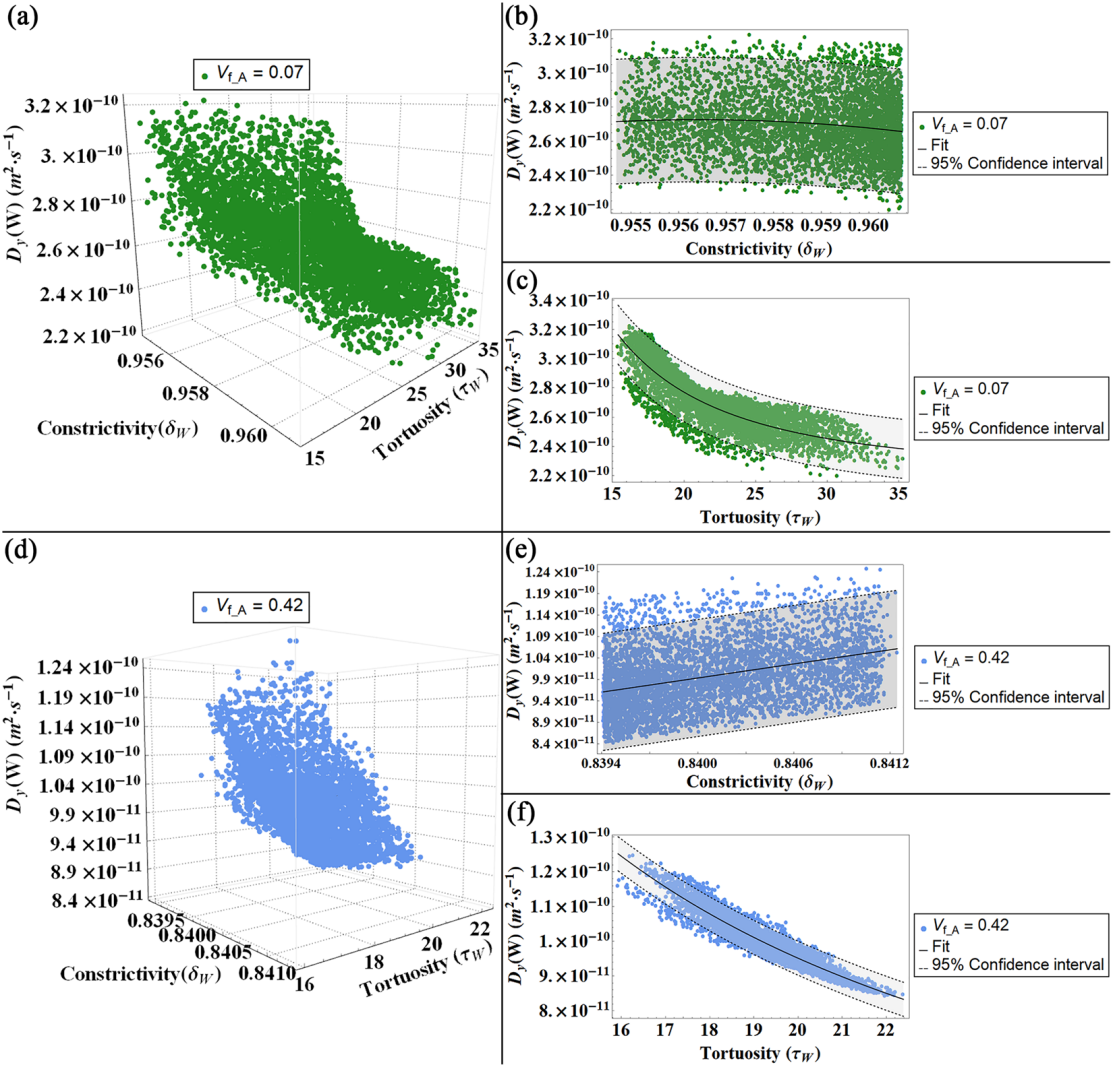
Diffusion coefficient (D_W_) for a flow parallel to the width direction (W) represented in function of the constrictivity and tortuosity factors for both models, i.e. V_f_A_ = 0.07 (**a–c**) and V_f_A_ = 0.42 (**d,f**): in (**a,d**) 3-D plot of the diffusivity D_W_ versus constrictivity and tortuosity. In (**b,e**) 2-D plot of D_W_ versus the constrictivity and in (**c,f**) 2-D plot of D_W_ versus the tortuosity. The colour bands in the 2-D plots indicates the Confidence Interval at 95 percent.

**Figure 8:**
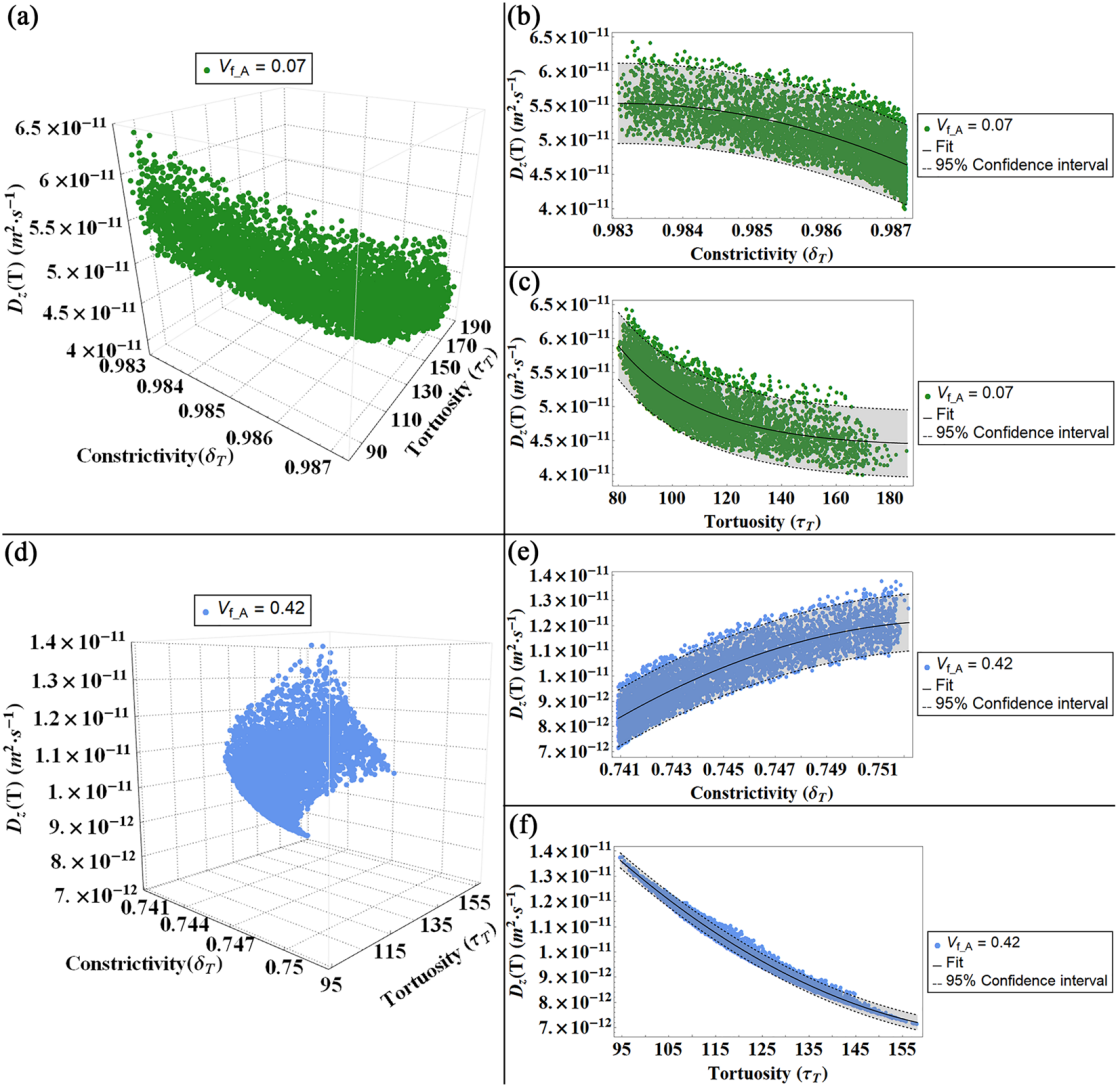
Diffusion coefficient (D_T_) for a flow parallel to the thickness direction (T) represented in function of the constrictivity and tortuosity factors for both models, i.e. V_f_A_ = 0.07 (**a–c**) and V_f_A_ = 0.42 (**d,f**): in (**a,d**) 3-D plot of the diffusivity D_T_ versus constrictivity and tortuosity. In (**b,e**) 2-D plot of D_T_ versus the constrictivity and in (**c,f**) 2-D plot of D_T_ versus the tortuosity. The colour bands in the 2-D plots indicates the Confidence Interval at 95 percent.

Subsequently, we represent the values of the diffusion coefficient in function of the tortuosity and the constrictivity calculated for flows parallel to the longitudinal (Fig. 6), width (Fig. 7) and thickness (Fig. 8) directions for both degrees of V_f_A_ = 0.07 and V_f_A_ = 0.42.

The outcomes of the intermediate level, i.e. V_f_A_ = 0.32, are reported in the Supplementary Figs S1–S3. We compared the behaviour of this mineral VF with the previous outcomes^28^ concerning a physiological mineralization degree.

## Discussions

It has been noticed that the water flux significantly influences the provision of nutrients and the interaction between mineral and proteins constituents^27^, enhancing the strength of the collagen apatite matrix^1^. Moreover, since the signalling functions have a fundamental role in bone remodelling^4,48^, an alteration of the diffusion properties could contribute to an abnormal development of the tissue.

In this study, we investigate the influence of geometric factors, i.e. tortuosity and constrictivity, on the water diffusion coefficient in the nanostructure of bone tissue for different degrees of mineral VF. We also highlight the dependence of the tortuosity and constrictivity on the mineral aspect ratio that characterizes each VF and on the arrangement of the constituent elements of the mineralized collagen fibrils. Water diffusion within bone nanostructure has been already analysed by experimental and numerical investigations^18,28^ and pronounced anisotropy of the structure has been revealed. As a consequence of this spatial feature, in the study of Marinozzi *et al*.^18^ the diffusion coefficient along the longitudinal axis of the trabecula (D_L_) is found to be greater with one order of magnitude than the coefficient along the width direction (D_W_) and with two orders of magnitude than the coefficient in the thickness direction (D_T_). The outcomes of the present study are consistent with previous investigations^3,18,28^ and offers additional information with regard to the relationship between the structural anisotropy and mass transport properties.

Overall, the results indicate that the variation of the mineral VF influences the values of tortuosity, constrictivity and diffusivity. It is worth pointing out that the order of magnitude of the ratio between the diffusion coefficient along the directions of CS_T_ is conserved for all the degrees of mineralization considered. For high apatite, V_f_A_ = 0.42, the combined effect of the tortuosity and constrictivity for all the principal flow directions investigated corresponds to a decrease of the diffusion coefficient with respect to the physiological values achieved previously by Marinozzi *et al*.^18^. Conversely, the low apatite, V_f_A_ = 0.07, is characterized by slightly higher values of the diffusion coefficient in comparison to the diffusivity investigated in physiological conditions^18^. In addition, the intermediate condition of mineral, V_f_A_ = 0.32, shows outcomes in agreement with the previous study^18^.

Figures 6–8 illustrate the dependence of the diffusion coefficient on the constrictivity and tortuosity factors. According to its definition (Eq. (6), see Methods section for details), the constrictivity assumes values in the range 0–1, where the maximum value represents the absence of variations in the section of the passageway of the water molecule. Conversely, the adopted definition for the tortuosity (Eq. (3), see Methods section for details) leads to values greater than 1. This observation implies that the presence of mineral increases the path length of the fluid between two points with respect to the shortest distance between the points in a homogeneous medium^32,49^. For a fixed mineral VF, the variations of the tortuosity and constrictivity and, subsequently, those of the effective diffusion coefficient are mainly due to changes in the platelets orientation with respect to the CS_T_. The influence of the apatite inclination relative to the trabecula main axes on the diffusion coefficient has been already discussed in a previous study^28^, whilst in this paper we focus on the relationship between the structural parameters and the diffusion coefficient.

For a parallel flow to the longitudinal direction (Fig. 6), the effective diffusion coefficient is slightly minor than the diffusivity in free space, i.e. D_0_ = 2.66·10^−9^ (m^2^·s^−1^)^29^. In fact, in this direction, the topology of the model is characterized by negligible obstacles. It should be noted that the constrictivity values have variations only in the third decimal place, namely for the low VF model δ_L_ ${\rm{\epsilon }}$ [0.954; 0.961], while for the high VF model δ_L_ ${\rm{\epsilon }}$ [0.839; 0.842]. Increasing the level of mineral VF, the variation of the platelets aspect ratio and the distance between mineral crystals leads to a decrement of the constrictivity values, and therefore to a slightly marked bottleneck effect on the diffusion coefficient.

For both levels of mineral VF, the tortuosity achieves values in a narrow range close to 1, namely for the low VF model τ_L_ ${\rm{\epsilon }}$ [1.20; 1.35], while for the high VF model τ_L_ ${\rm{\epsilon }}$ [1.13; 1.23]. These outcomes imply that, independently from the range of inclinations of the apatite platelets, in the longitudinal direction, the diffusing particle frequently does not encounter geometrical obstructions in terms of the presence of barriers that could extend significantly the length of the streamline with respect to the Euclidean distance between the path extremes. Moreover, increasing the platelets dimensions and reducing the distances between the mineral, as it occurs in the high VF condition, leads to a decrease of the tortuosity. Since the same set of values for the unit cell inclination was considered in the numerical simulations of all degrees of VF, this reduction of the tortuosity in the high VF model is justified by the increment of continuous straight paths with respect to the length of possible path deviations.

For a flow parallel to the width direction, a noticeable dependence of the diffusion coefficient D_W_ on the tortuosity could be highlighted (Fig. 7). Along this direction, the diffusing particles are more frequently interrupted by obstacles, i.e. the apatite minerals, and, therefore the tortuosity is higher with an order of magnitude in comparison with the tortuous factor in the longitudinal direction. Conversely, the constrictivity factor shows a minor impact on the diffusivity trend. For each mineral VF, the variations of the constrictivity factor due to the different Gaussian PDFs considered for the mineral inclinations with respect to the CS_T_, are extremely smaller, with changes in the third decimal place. The narrow interval suggests that the constrictivity is less influenced by the inclinations of the mineral platelets, meanwhile it exhibits a dependence on the degree of mineralization, assuming higher values in the low VF case.

In the thickness direction, the diffusion coefficient shows the minimum values among the other directions, with two orders of magnitude minor than D_L_ (Fig. 8). The diffusivity along the thickness direction allows to properly highlight the major influence of the tortuosity on controlling the effective transport properties. In this condition, the tortuosity factor achieves higher values than in the longitudinal case with roughly two orders of magnitude. This is an expected behaviour since in the thickness direction, the diffusion pathways are the most complex, due to the spatial arrangement of the mineral platelets. Conversely, the constrictivity is characterized by narrow intervals, with variations in the third decimal place consequent to the different Gaussian PDFs considered for the apatite inclination. It is worth pointing out that for flow parallel to the thickness direction, the constrictivity is substantially influenced by variations of VF. In fact, in the high mineral VF model, changes in the cross section of the pores due to reductions of the distances between the mineral platelets results in a remarkable diminution of the constrictivity factor in comparison to the low VF condition.

In this study, we have predicted the diffusion coefficient within the unit cell of bone nanostructure relating it to the internal geometry. The different mineral aspect ratio, achieved for the degrees of mineralization analysed, i.e. V_f_A_ = 0.07 and V_f_A_ = 0.42, influences significantly the three factors that interact with the self-diffusion coefficient, i.e. tortuosity, constrictivity and porosity. The outcomes confirm that the tortuosity and constrictivity factors are mostly governed by the geometry of the structure. A growth of the mineral VF corresponds to a mineral matrix more densely embedded in the collagen fibrils which leads to smaller values of constrictivity, thus to a more marked bottleneck effect. Overall, the constrictivity factor shows modest variations with respect to the platelets arrangement for fixed VF and with respect to the concentration gradient. Conversely, the tortuosity displays a significant variation for the different flow directions considered. For concentration gradients parallel to the width and thickness directions, the tortuosity is higher with one and two orders of magnitude respectively, in comparison with the tortuosity in the longitudinal direction. It emerges that the tortuosity is significantly influenced by the anisotropy of the medium and it is, therefore, the main parameter controlling the effective diffusion coefficient.

These outcomes could provide also further insights into bone nanostructure geometry. Moreover, a critical point revealed by this study is referred to the average dimensions adopted for the apatite platelets in each level of VF analysed. A significant improvement in the numerical analysis performed consists in taking into account the variation of the platelets dimensions within the range present in literature. As highlighted in the Introduction, the dimensions of the platelets are well defined in literature^9,13^, whilst the distances between the mineral crystals (e.g. a_W_) are scarcely characterized. In the Supplementary Table S2 we report the average dimensions assumed for each model. We considered that, on average, an increased level of VF corresponds to higher dimensions of the apatite crystals and reduced distances between the platelets. In the present model, for different degrees of VF, the width of the apatite platelets has the major variation among all the other geometrical parameters, e.g. in the high VF condition the average width is roughly three times higher than the average width that characterizes the low apatite VF case^13^. Conversely, the increment of the mineral in the longitudinal direction is characterized by a minor factor, since the length of the apatite increases on average from 70 nm in the low VF model to 105 nm in the high VF condition. Minimal variations are considered also for the crystal thickness. The distance between the platelets in the longitudinal direction has geometric constraints, determined by the staggered pattern with axial period D = 67 nm. The distance along the thickness direction is assumed on the same order of magnitude as the apatite thickness^15,23^. In addition, the distance in the width direction that allows to achieve the mineral VF of interest is on the same order of magnitude as the minimum width dimension of the apatite, i.e. 10 nm^13^. The good agreement between the effective diffusivities obtained in this study and the literature data^18,30^ confirms the suitability of the sets of geometric dimensions considered for the mineralized collagen fibrils. Moreover, the corroboration of the mass transport coefficients represents also a validation of the arrangement of the mineral platelets along the width and thickness directions assumed in the model.

The analysis focuses on extreme values of the interval that describes the mineral content of the human bone matrix^20,21^, i.e. V_f_A_ = 0.07 and V_f_A_ = 0.42. This approach allows to investigate the mass transport behaviour in conditions that represent significant alterations of the mineralization level. For instance, a small amount of apatite crystallites is encountered in the pathological condition of osteomalacia^50^, while high mineral VFs are characteristic of the osteoporotic tissue^50,51^. However, the number of experimental and computational studies that evaluate variations in altered VFs structures are limited. In fact, the structural assumptions adopted for the low VF condition are based on computational studies^23^, since, to the best of our knowledge, experimental characterization at the nanoscale has not been undertaken. Conversely, the hypotheses relative to the apatite arrangement in the high VF case are supported by SAXS and TEM investigations^13,50^. The latter studies highlighted that the spatial structure remains invariant with respect to a physiological degree of VF, while modifications occur in terms of platelets size and interspacing^52^. In addition, the outcomes of the high VF condition, namely the decrease of the diffusion coefficient with respect to the physiological values achieved by Marinozzi *et al*.^18^, are in agreement with the experimental study of Roschger *et al*.^20^ In fact, they observed that in the osteoporotic disease, free water in the matrix is gradually replaced by mineral, and the mineralization density is approaching a plateau value, corresponding to the percolation threshold^20^. However, more research investigation with regard to the nanomorphology of the mineralized collagen fibril is required to fully extend the numerical approach presented in this work also to pathological situations.

In conclusion, we improved a previous computational model of bone nanostructure^28^ by considering more realistic fluctuations of the geometrical parameters that define the mineralized collagen fibrils. We applied the concepts of tortuosity and constrictivity in the diffusion process within the bone nanostructure and analysed their influence on the effective diffusion coefficient of water. However, there are several limitations to acknowledge. The model currently considers a limited unit cell composed of three apatite platelets. This is known as a simplification and, in perspective, a major number of mineral crystals should be assembled in order to obtain more accurate prediction of the diffusion coefficient and also to mimic a higher hierarchical level of bone structure, e.g. trabecula.

It should also be mentioned that an ulterior limitation of this study consists in the axial period of 67 nm set for the staggered pattern of mineral apatite in the longitudinal direction. Whilst the staggered arrangement is well established^11^ and corroborated by experimental studies^9,46^, the dimension of the period D is still under investigation^53^. It is worth investigating an optimal size which could provide the proper behaviour of the mineralized collagen fibril in terms of mass transport and mechanical properties. In fact, this longitudinal periodicity of the structure influences the load transfer between the collagen matrix and the apatite platelets and optimizes the mechanical properties of the composite^10,54^.

Despite these limitations, it may be observed that the computational model provides a good prediction of the diffusion coefficient. This model approach may offer a valuable foundation for future investigation in order to further understand the structure and mechanical behaviour of the mineralized collagen fibrils. Moreover, an optimization of the computational model could be a valuable support for predictive studies of the mechanical properties of biocomposite that mimic bone tissue^55^ or that could improve bone behaviour like biosensors based on ZnO nanomaterials^56,57^.

## Methods

This work extends a previous computational study of water diffusion coefficient within bone nanostructure^28^. Enhancements and crucial aspects are summarised in this section while the equations implemented in the numerical model are reported as Supplementary Eqs S1–S54.

### Geometry model overview

The analysis is based on a 3-D unit cell of the mineralized collagen fibril (Fig. 1). We consider three apatite VFs, that represent low (V_f_A_ = 0.07), intermediate (V_f_A_ = 0.32) and high (V_f_A_ = 0.42) mineralized conditions. The mineral VF is obtained according to Eq. (1)^23^:

1 $${V}_{f\_A}=\frac{L\cdot W\cdot T}{(L+{a}_{L})\cdot (W+{a}_{W})\cdot (T+{a}_{T})}$$

where L, W, T are the length, width and thickness of the mineral platelet, a_L_, a_W_ and a_T_ are the distances between the platelets in the longitudinal, width and thickness direction (Fig. 3).

With respect to the previous model^28^, in this study we improved the 3D geometric model by considering all random values for the geometrical parameters involved in the characterization of the mineralized collagen fibril. These values were achieved by means of random extractions from Gaussian PDFs in the ranges indicated in literature^8,10,13,15,16^ (Supplementary Table S1). In particular, we assumed that the collagen diameter (d) range values spanning^8^ from 1.1 nm to 1.4 nm, while the distance between adjacent fibrils (a_c_) is in the range^10^ from 1 to 1.5 nm. Furthermore, in agreement with experimental analysis^13^, we considered for the apatite thickness (T) a range of values between 2.5 nm to 5 nm. Subsequently, these values are used as input parameters in the Monte Carlo computational analysis.

We assume that the mineral platelets and the collagen fibril composing a functional unit cell maintain their reciprocal parallelism (Fig. 1) independently of the cell inclination with respect to a global coordinate system (CS_T_) fixed to the axes of the single trabeculae (Length L, Width W and Thickness T)^28^. We also consider that the orientation of the unit cell, i.e. the tern (θ_LT_, θ_WT_, θ_LW_), is on average parallel to the axes of CS_T_. Thus, the numerical values for the tern (θ_LT_, θ_WT_, θ_LW_) were set from Gaussian PDFs identified by mean zero degrees and variable values of standard deviation (σ)^14,15^. In order to have a finite angle domain for each inclination θ_LT_, θ_WT_, θ_LW_, numerical simulations were carried out with truncated Gaussian distributions in the interval [−2σ; +2σ].

### Diffusion coefficient

The mineralized collagen fibril is characterized as a composite porous material. Thus, the effective diffusivity (D_eff_) is achieved introducing the tortuosity (τ), constrictivity (δ) and porosity factors (φ)^32,33^:

2 $$\begin{array}{c}{D}_{eff}({\theta }_{LT},{\theta }_{LW},{\theta }_{WT},L,W,T,{a}_{L},{a}_{W},{a}_{T})\\ \,=\,{D}_{0}\cdot \frac{\delta ({\theta }_{LT},{\theta }_{LW},{\theta }_{WT},L,W,T,{a}_{L},{a}_{W},{a}_{T})\cdot \phi (L,W,T,{a}_{L},{a}_{W},{a}_{T})}{\tau ({\theta }_{LT},{\theta }_{LW},{\theta }_{WT},L,W,T,{a}_{L},{a}_{W},{a}_{T})}\end{array}$$

In a homogeneous medium, the diffusion coefficient of water is D_0_ = 2.66 10^−9^ m^2^·s^−1^ at 27 °C^29^.

### Tortuosity

The tortuosity factor (τ) is defined as the square ratio between the effective path length (*l*_*i*_) of the sinuous streamline and the Euclidean straight path, (*l*_*i_E*_) in the direction of flow, between the path extremes^31,32,40^, i.e.:

3 $${\rm{\tau }}={(\frac{{l}_{i}}{{l}_{i\_E}})}^{2}$$

The mineral matrix within the collagen fibrils is assumed as an impermeable obstacle to the water diffusion. Therefore, the tortuosity is influenced by the arrangement of the collagen-apatite matrix and flow direction. The numerical simulations were implemented assuming that a concentration gradient exists along one of the three axes of the CS_T_ at a time and the pore space allows the crossing of a single molecule of water at a time. The assessment of the tortuosity was performed by analysing separately the contribution of the mineral and collagen matrix, respectively. Subsequently, the overall tortuosity is obtained applying Eq. (8) reported in the study of Bini *et al*.^28^.

The tortuosity in the mineral matrix is determined with a geometrical approach. Taking into account the direction of the concentration gradient, we considered probable flow paths inside the unit cell in all the three main planes, i.e. LT, LW and WT (Figs 2–4, green dotted lines). The resulting trajectories lengths (*l*_*i*_) are achieved through the Supplementary Eqs S1–S36. Each equation is function of the dimensions of the mineral platelets, the spacing between the minerals and the inclination (θ_LT_, θ_WT_, θ_LW_) of the apatite with respect to the CS_T_. The denominator of Eq. (3) represents the straight-line distance in the direction of flow that connects the initial and final point of the tortuous flow path (Figs 2–4, red continuous lines). Supplementary Eqs S1–S36 assess also the straight-line distance (*l*_*i_E*_).

To consider the collagen contribution to the tortuosity factor, we analysed cross sections of the geometrical model parallel to the LT, LW and WT planes of CS_T_ (Figs 2–4). Since the collagen is inclined with respect to the CS_T_, the cross sections have elliptical shape. We assumed that the water molecule, given the high density of the collagen fibrils and their arrangement, performs tangent trajectories to the fibril characterized by a length equal to the semi-perimeter of the ellipse. Whether the ellipse is expressed as a parametric curve, i.e. $\{x(t)=a\cdot \,\cos (t),y(t)=b\cdot \,\sin (t)\}$, its semi-perimeter can be calculated through the elliptic integral of second kind^58^:

4 $$L(t)={\int }_{{t}_{\min }}^{{t}_{\max }}\sqrt{x^{\prime} {(t)}^{2}+y^{\prime} {(t)}^{2}}dt$$

where **a** is the semi-major axis of the ellipse, **b** is the semi-minor axis of the ellipse and **t**_**min**_, **t**_**max**_ are the extreme angles of the arc considered. The semi-axes of the ellipse are determined according to Supplementary Eqs S37–S48.

In the case of the collagen matrix, the Euclidean distance, *l*_*i_E*_, is represented by the major axis of the ellipse.

### Constrictivity

The constrictivity δ ${\rm{\epsilon }}$ [0, 1] represents the hindrance to which the fluid is subject travelling through pores of varying cross section^33,36^. It becomes important only if the size of the solute, i.e. water molecule, is comparable to the dimensions of the pores. Several studies^59,60^ evaluate δ as a function of the ratio of the maximum and minimum cross section for different pore size structures:

5 $$\delta =\frac{\sqrt{\max \,cross\,\text{sec}tion\cdot \,\min \,cross\,\text{sec}tion}}{mean\,\,cross\,\text{sec}tion}$$

Variations of the width of the passageway in the collagen-mineral matrix are identified by comparing the distance between the collagen fibrils and the spacing between collagen and apatite (Fig. 5). These distances are influenced by the dimensions of the matrix and the inclination with respect to the CS_T_. The adaptation of Eq. (5) for each plane of our model is represented by the Supplementary Eqs S49–S54.

### Porosity

The porosity is considered independent from the inclination of the functional unit cell and is calculated by Eq. (5) reported in the previous study^28^.

### Numerical simulation

Montecarlo method (Mathematica 10, Wolfram, Oxfordshire, UK) is used to assess the tortuosity, constrictivity and diffusion coefficient along the main directions of the CS_T_ for each percentage of VF. The computational algorithm developed for a concentration gradient parallel to one of the main axes of the CS_T_ can be summarized as follows:

- Random extraction of the standard deviations (σ_LT_, σ_LW_, σ_WT_) that define the Gaussian PDFs;
- Fixed a value for σ_LT_, σ_LW_ and σ_WT_, respectively, random extraction of the orientation angles θ_LT_, θ_LW_, θ_WT_ from the corresponding Gaussian PDFs are performed;
- Random extraction of the geometric parameters that characterize the apatite matrix, i.e. L, W, T, a_T_, a_W_, and the collagen fibrils, i.e. d, a_c_, from the corresponding Gaussian PDFs.
- Calculation of the constrictivity and the tortuosity through the Supplementary Eqs S1–S54 based on Eqs (5) and (3), respectively.
- Calculation of the effective diffusion coefficient in accordance to Eq. (2).
- For a fixed concentration gradient, the geometrical approach implemented for the assessment of the constrictivity and tortuosity allow to achieve information about the previous two structural factors and the effective diffusion coefficient from two planes. Therefore, an average between the two values resulting from the different planes is performed.
- Repeating steps from (b) to (f) until completing N realizations equal to 5000.
- Calculation of the mean of means of the constrictivity, tortuosity and effective diffusion coefficient respectively, corresponding to the Gaussian PDF considered for the mineral inclinations.

Therefore, in Figs 6–8 we represent the mean of means of the effective diffusion coefficients in function of the mean of means of the tortuosity and the mean of means of the constrictivity. In order to obtain the outcomes reported in Figs 6–8, we repeat the previous steps (a)–(h) for 5000 different Gaussian PDFs, performing overall up to 25·10^6^ of iterations. Whilst in model^28^ the results show as the apatite inclination affects the effective diffusion coefficient for 100 different Gaussian PDFs, performing overall up to 10^4^ iterations.

### Statistical analysis

We performed a nonlinear fit on the mean of means of the diffusivity corresponding to each diffusion gradient in function of the tortuosity and constrictivity factors, respectively. We also reported a confidence interval of 95 percent that provides the interval for predicting the diffusivity mean of means at fixed values of the predictors, i.e. mean of means of tortuosity and mean of means of constrictivity. It is calculated as follows:

6 $${D}_{fitting}\pm k\cdot \sqrt{{\sigma }^{2}+\sum }$$

where **D**_**fitting**_ is the fitting value of the diffusivity mean of means, **k** = 1.96044 is the coverage factor, **σ**^**2**^ is the estimated error variance and **Σ** is the estimated covariance matrix for the parameters. The estimated error variance is calculated as:

7 $${\sigma }^{2}=\frac{\sum _{i}{({D}_{i}-{D}_{fitting})}^{2}}{N-1}$$

The estimated covariance matrix is obtained as:

8 $$\Sigma ={\hat{\sigma }}^{2}\cdot {({X}^{T}\cdot W\cdot X)}^{-1}$$

where ${\hat{{\boldsymbol{\sigma }}}}^{2}$ is the variance estimate, **X** is the design matrix and **W** is the diagonal matrix of weights.

## Supplementary information

SUPPLEMENTARY INFORMATION

## Data Availability

The datasets generated and/or analysed during the current study are available from the corresponding author upon reasonable request.
