# Supplementary material for: A 3D Model of the Effect of Tortuosity and Constrictivity on the Diffusion in Mineralized Collagen Fibril
Source: Sci Rep. 2019 Feb 25;9:2658. doi: 10.1038/s41598-019-39297-w (PMC6389916; doi:10.1038/s41598-019-39297-w)
Supplement: Supplementary file 1 — SUPPLEMENTARY INFORMATION [file 41598_2019_39297_MOESM1_ESM.pdf]

A 3D Model of the Effect of Tortuosity and Constrictivity on the Diffusion in Mineralized Collagen Fibril

Fabiano Bini[1*], Andrada Pica[1], Andrea Marinozzi[2], Franco Marinozzi[1]

[1]Department of Mechanical and Aerospace Engineering, "Sapienza" University of Rome, via Eudossiana, 18 – 00184 Rome, Italy

[2]Orthopedy and Traumatology Area, "Campus Bio-Medico" University, via Alvaro del Portillo, 200-00128 Rome, Italy

Address correspondence to Fabiano Bini, Department of Mechanical and Aerospace Engineering, "Sapienza" University of Rome, via Eudossiana, 18 – 00184 Rome, Italy.

Corresponding author

fabiano.bini@uniroma1.it tel. 00390644585143 – fax 00390644585250.

Supplementary equations

The tortuosity is assessed by means of a geometrical 3D model. For each plane (LT, WT and LW plane), we considered flows parallel to the main orthogonal directions that characterize the plane. According to the geometry of the structure, in each plane different streamlines are assumed in function of the concentration gradient. Equations that allow to calculate the length of the tortuous streamlines within the mineral matrix (l_i) and the Euclidean distance (l_{i_E}) between the path extremes are presented below. The lengths l_i and l_{i_E} are then used in Eq. (3).

The streamlines considered in each of the three planes (LT, WT and LW plane) are characterized by an interval of existence depending on the angle that describes the inclination of the platelets in the specific plane, i.e. θ_{LT} in the LT plane, θ_{LW} in the LW plane and θ_{WT} in the WT plane.

For each direction of the concentration gradient, we report the main equations that are applied in the calculation of the tortuosity factor.

Concentration gradient parallel to the longitudinal axis (L) of the CS$_T$

Equations that describe the streamlines in the LT plane for a flow parallel to the longitudinal axis

$$\begin{cases} \boldsymbol{l}_1 = \dfrac{(\boldsymbol{T}+\boldsymbol{a_T})\cdot\cos(\boldsymbol{\theta_{WT}})}{\sin(\boldsymbol{\theta_{LT}})} + 0.5\cdot(\boldsymbol{L}+\boldsymbol{a_L})\cdot\cos(\boldsymbol{\theta_{LW}})\cdot\cos(\boldsymbol{\theta_{LT}}) + \\ -(\boldsymbol{T}+\boldsymbol{a_T})\cdot\cos(\boldsymbol{\theta_{WT}})\cdot\cot(\boldsymbol{\theta_{LT}}) + \boldsymbol{T}\cdot\cos(\boldsymbol{\theta_{WT}})\cdot\cos(\boldsymbol{\theta_{LT}}) \\ \boldsymbol{l}_{1\_E} = \left[0.5\cdot(\boldsymbol{L}+\boldsymbol{a_L})\cdot\cos(\boldsymbol{\theta_{LW}})\right]\cdot\cos(\boldsymbol{\theta_{LT}}) + \boldsymbol{a_T}\cdot\cos(\boldsymbol{\theta_{WT}})\cdot\sin(\boldsymbol{\theta_{LT}}) \\ \arctan\left(\dfrac{\boldsymbol{T}\cdot\cos(\boldsymbol{\theta_{WT}})}{\boldsymbol{a_L}\cdot\cos(\boldsymbol{\theta_{LW}})}\right) \le \boldsymbol{\theta_{LT}} \le \dfrac{\pi}{2} \end{cases} \tag{S1}$$

$$\begin{cases} \boldsymbol{l}_2 = (\boldsymbol{T}+\boldsymbol{a_T})\cdot\cos(\boldsymbol{\theta_{WT}})\cdot\cos(\boldsymbol{\theta_{LT}}) + \dfrac{0.5\cdot(\boldsymbol{L}+\boldsymbol{a_L})\cdot\cos(\boldsymbol{\theta_{LW}})}{\cos(\boldsymbol{\theta_{LT}})} + \boldsymbol{T}\cdot\cos(\boldsymbol{\theta_{WT}})\cdot\cos(\boldsymbol{\theta_{LT}}) \\ -2\cdot\left[0.5\cdot(\boldsymbol{L}+\boldsymbol{a_L})\cdot\cos(\boldsymbol{\theta_{LW}})\cdot\tan(\boldsymbol{\theta_{LT}}) - \boldsymbol{a_T}\cdot\cos(\boldsymbol{\theta_{WT}})\cdot\cos(\boldsymbol{\theta_{LT}})\right] \\ \boldsymbol{l}_{2\_E} = (\boldsymbol{L}+\boldsymbol{a_L})\cdot\cos(\boldsymbol{\theta_{LW}})\cdot\cos(\boldsymbol{\theta_{LT}}) + (2\cdot\boldsymbol{T}+\boldsymbol{a_T})\cdot\cos(\boldsymbol{\theta_{WT}})\cdot\sin(\boldsymbol{\theta_{LT}}) \\ \arctan\left(\dfrac{\boldsymbol{a_T}\cdot\cos(\boldsymbol{\theta_{WT}})}{0.5\cdot(\boldsymbol{L}-\boldsymbol{a_L})\cdot\cos(\boldsymbol{\theta_{LW}})}\right) \le \boldsymbol{\theta_{LT}} \le \arctan\left(\dfrac{\boldsymbol{a_T}\cdot\cos(\boldsymbol{\theta_{WT}})}{0.5\cdot(\boldsymbol{L}+\boldsymbol{a_L})\cdot\cos(\boldsymbol{\theta_{LW}})}\right) \end{cases} \tag{S2}$$

$$\begin{cases} \boldsymbol{l}_3 = \boldsymbol{T}\cdot\cos(\boldsymbol{\theta_{WT}})\cdot\cos(\boldsymbol{\theta_{LT}}) + \dfrac{\boldsymbol{a_T}\cdot\cos(\boldsymbol{\theta_{WT}})}{\sin(\boldsymbol{\theta_{LT}})} + \\ +2\cdot\left(\left(0.5\cdot(\boldsymbol{L}-\boldsymbol{a_L})\cdot\cos(\boldsymbol{\theta_{LW}})\cdot\cos(\boldsymbol{\theta_{LT}})\right) - \boldsymbol{a_T}\cdot\cos(\boldsymbol{\theta_{WT}})\cdot\cot(\boldsymbol{\theta_{LT}})\right) + \\ +2\cdot\left(\dfrac{\boldsymbol{a_L}\cdot\cos(\boldsymbol{\theta_{LW}})}{\cos(\boldsymbol{\theta_{LT}})} + \boldsymbol{T}\cdot\cos(\boldsymbol{\theta_{WT}})\cdot\cos(\boldsymbol{\theta_{LT}}) - \boldsymbol{a_L}\cdot\cos(\boldsymbol{\theta_{LW}})\cdot\tan(\boldsymbol{\theta_{LT}})\right) + \\ +\dfrac{\boldsymbol{a_T}\cdot\cos(\boldsymbol{\theta_{WT}})}{\sin(\boldsymbol{\theta_{LT}})} + \boldsymbol{T}\cdot\cos(\boldsymbol{\theta_{WT}})\cdot\cos(\boldsymbol{\theta_{LT}}) \\ \boldsymbol{l}_{3\_E} = (\boldsymbol{L}+\boldsymbol{a_L})\cdot\cos(\boldsymbol{\theta_{LW}})\cdot\cos(\boldsymbol{\theta_{LT}}) + 2\cdot(\boldsymbol{T}+\boldsymbol{a_T})\cdot\cos(\boldsymbol{\theta_{WT}})\cdot\sin(\boldsymbol{\theta_{LT}}) \\ \arctan\left(\dfrac{\boldsymbol{a_T}\cdot\cos(\boldsymbol{\theta_{WT}})}{0.5\cdot(\boldsymbol{L}-\boldsymbol{a_L})\cdot\cos(\boldsymbol{\theta_{LW}})}\right) \le \boldsymbol{\theta_{LT}} \le \dfrac{\pi}{2} \end{cases} \tag{S3}$$

$$\begin{cases} \boldsymbol{l}_4 = \dfrac{\boldsymbol{a}_L \cdot \cos(\boldsymbol{\theta}_{LW})}{\cos(\boldsymbol{\theta}_{LT})} + \boldsymbol{T} \cdot \cos(\boldsymbol{\theta}_{WT}) \cdot \cos(\boldsymbol{\theta}_{LT}) - \boldsymbol{a}_L \cdot \cos(\boldsymbol{\theta}_{LW}) \cdot \tan(\boldsymbol{\theta}_{LT}) + \\ + \boldsymbol{L} \cdot \cos(\boldsymbol{\theta}_{LW}) \cdot \cos(\boldsymbol{\theta}_{LT}) + \boldsymbol{a}_T \cdot \cos(\boldsymbol{\theta}_{WT}) \cdot \cos(\boldsymbol{\theta}_{WT}) \\ \boldsymbol{l}_{4\_E} = (\boldsymbol{L} + \boldsymbol{a}_L) \cdot \cos(\boldsymbol{\theta}_{LW}) \cdot \cos(\boldsymbol{\theta}_{LT}) + (\boldsymbol{T} - \boldsymbol{a}_T) \cdot \cos(\boldsymbol{\theta}_{WT}) \cdot \sin(\boldsymbol{\theta}_{LT}) \\ 0 \le \boldsymbol{\theta}_{LT} \le \arctan\left(\dfrac{\boldsymbol{T} \cdot \cos(\boldsymbol{\theta}_{WT})}{\boldsymbol{a}_L \cdot \cos(\boldsymbol{\theta}_{LW})} \right) \end{cases} \tag{S4}$$

$$\begin{cases} \boldsymbol{l}_5 = \boldsymbol{T} \cdot \cos(\boldsymbol{\theta}_{WT}) \cdot \cos(\boldsymbol{\theta}_{LT}) + 2 \cdot \dfrac{\boldsymbol{a}_T \cdot \cos(\boldsymbol{\theta}_{WT})}{\sin(\boldsymbol{\theta}_{LT})} + \\ +2 \cdot \left(\left(0.5 \cdot (\boldsymbol{L} - \boldsymbol{a}_L) \cdot \cos(\boldsymbol{\theta}_{LW}) \cdot \cos(\boldsymbol{\theta}_{LT}) \right) - \boldsymbol{a}_T \cdot \cos(\boldsymbol{\theta}_{WT}) \cdot \cot(\boldsymbol{\theta}_{LT}) \right) + \\ +2 \cdot \left(\dfrac{\boldsymbol{a}_L \cdot \cos(\boldsymbol{\theta}_{LW})}{\cos(\boldsymbol{\theta}_{LT})} + \boldsymbol{T} \cdot \cos(\boldsymbol{\theta}_{WT}) \cdot \cos(\boldsymbol{\theta}_{LT}) - \boldsymbol{a}_L \cdot \cos(\boldsymbol{\theta}_{LW}) \cdot \tan(\boldsymbol{\theta}_{LT}) \right) + \\ +(\boldsymbol{T} + \boldsymbol{a}_T) \cdot \cos(\boldsymbol{\theta}_{WT}) \cdot \cos(\boldsymbol{\theta}_{LT}) + \left(0.5 \cdot (\boldsymbol{L} - \boldsymbol{a}_L) \cdot \cos(\boldsymbol{\theta}_{LW}) \cdot \cos(\boldsymbol{\theta}_{LT}) \right) \\ \boldsymbol{l}_{5\_E} = (1.5 \cdot \boldsymbol{L} + 0.5 \cdot \boldsymbol{a}_L) \cdot \cos(\boldsymbol{\theta}_{LW}) \cdot \cos(\boldsymbol{\theta}_{LT}) + (2 \cdot \boldsymbol{T} + \boldsymbol{a}_T) \cdot \cos(\boldsymbol{\theta}_{WT}) \cdot \sin(\boldsymbol{\theta}_{LT}) \\ \arctan\left(\dfrac{\boldsymbol{a}_T \cdot \cos(\boldsymbol{\theta}_{WT})}{0.5 \cdot (\boldsymbol{L} - \boldsymbol{a}_L) \cdot \cos(\boldsymbol{\theta}_{LW})} \right) \le \boldsymbol{\theta}_{LT} \le \dfrac{\boldsymbol{\pi}}{2} \end{cases} \tag{S5}$$

$$\begin{cases} \boldsymbol{l}_6 = \boldsymbol{T} \cdot \cos(\boldsymbol{\theta}_{WT}) \cdot \cos(\boldsymbol{\theta}_{LT}) + 2 \cdot \dfrac{\boldsymbol{a}_T \cdot \cos(\boldsymbol{\theta}_{WT})}{\sin(\boldsymbol{\theta}_{LT})} + \\ +2 \cdot \left(\left(0.5 \cdot (\boldsymbol{L} - \boldsymbol{a}_L) \cdot \cos(\boldsymbol{\theta}_{LW}) \cdot \cos(\boldsymbol{\theta}_{LT}) \right) - \boldsymbol{a}_T \cdot \cos(\boldsymbol{\theta}_{WT}) \cdot \cot(\boldsymbol{\theta}_{LT}) \right) + \\ +2 \cdot \left(\dfrac{\boldsymbol{a}_L \cdot \cos(\boldsymbol{\theta}_{LW})}{\cos(\boldsymbol{\theta}_{LT})} + \boldsymbol{T} \cdot \cos(\boldsymbol{\theta}_{WT}) \cdot \cos(\boldsymbol{\theta}_{LT}) - \boldsymbol{a}_L \cdot \cos(\boldsymbol{\theta}_{LW}) \cdot \tan(\boldsymbol{\theta}_{LT}) \right) + \\ +(\boldsymbol{T} + \boldsymbol{a}_T) \cdot \cos(\boldsymbol{\theta}_{WT}) \cdot \cos(\boldsymbol{\theta}_{LT}) \\ \boldsymbol{l}_{6\_E} = (\boldsymbol{L} + \boldsymbol{a}_L) \cdot \cos(\boldsymbol{\theta}_{LW}) \cdot \cos(\boldsymbol{\theta}_{LT}) + (2 \cdot \boldsymbol{T} + \boldsymbol{a}_T) \cdot \cos(\boldsymbol{\theta}_{WT}) \cdot \sin(\boldsymbol{\theta}_{LT}) \\ \arctan\left(\dfrac{\boldsymbol{a}_T \cdot \cos(\boldsymbol{\theta}_{WT})}{0.5 \cdot (\boldsymbol{L} - \boldsymbol{a}_L) \cdot \cos(\boldsymbol{\theta}_{LW})} \right) \le \boldsymbol{\theta}_{LT} \le \dfrac{\boldsymbol{\pi}}{2} \end{cases} \tag{S6}$$

Equations that describe streamlines in the LW plane for a flow parallel to the longitudinal axis

$$\begin{cases} l_7 = \dfrac{0.5 \cdot a_W \cdot \cos(\theta_{WT})}{\sin(\theta_{LW})} + (L + 2 \cdot a_L) \cdot \cos(\theta_{LT}) \cdot \cos(\theta_{LW}) - 0.5 \cdot a_W \cdot \cos(\theta_{WT}) \cdot \cot(\theta_{LW}) \\ l_{7\_E} = (L + 2 \cdot a_L) \cdot \cos(\theta_{LT}) \cdot \cos(\theta_{LW}) + (0.5 \cdot a_W) \cdot \cos(\theta_{WT}) \cdot \sin(\theta_{LW}) \\ \arctan\left(\dfrac{0.5 \cdot a_W \cdot \cos(\theta_{WT})}{a_L \cdot \cos(\theta_{LW})}\right) \le \theta_{LW} \le \arctan\left(\dfrac{0.5 \cdot a_W \cdot \cos(\theta_{WT})}{(L + 2 \cdot a_L) \cdot \cos(\theta_{LW})}\right) \end{cases} \tag{S7}$$

$$\begin{cases} l_8 = \dfrac{0.5 \cdot a_W \cdot \cos(\theta_{WT})}{\sin(\theta_{LW})} + (L + a_L) \cdot \cos(\theta_{LT}) \cdot \cos(\theta_{LW}) + \\ -0.5 \cdot a_W \cdot \cos(\theta_{WT}) \cdot \cot(\theta_{LW}) + a_W \cdot \cos(\theta_{WT}) \cdot \cos(\theta_{LW}) \\ l_{8\_E} = (L + a_L) \cdot \cos(\theta_{LT}) \cdot \cos(\theta_{LW}) + (0.5 \cdot a_W) \cdot \cos(\theta_{WT}) \cdot \sin(\theta_{LW}) \\ \arctan\left(\dfrac{0.5 \cdot a_W \cdot \cos(\theta_{WT})}{(L + a_L) \cdot \cos(\theta_{LW})}\right) \le \theta_{LW} \le \arctan\left(\dfrac{0.5 \cdot a_W \cdot \cos(\theta_{WT})}{a_L \cdot \cos(\theta_{LW})}\right) \end{cases} \tag{S8}$$

$$\begin{cases} l_9 = \dfrac{0.5 \cdot a_W \cdot \cos(\theta_{WT})}{\sin(\theta_{LW})} + (L + a_L) \cdot \cos(\theta_{LT}) \cdot \cos(\theta_{LW}) + \\ -0.5 \cdot a_W \cdot \cos(\theta_{WT}) \cdot \cot(\theta_{LW}) + a_L \cdot \cos(\theta_{LT}) \cdot \cos(\theta_{LW}) \\ l_{9\_E} = (L + 2 \cdot a_L) \cdot \cos(\theta_{LT}) \cdot \cos(\theta_{LW}) + (0.5 \cdot a_W) \cdot \cos(\theta_{WT}) \cdot \sin(\theta_{LW}) \\ \arctan\left(\dfrac{0.5 \cdot a_W \cdot \cos(\theta_{WT})}{(L + a_L) \cdot \cos(\theta_{LW})}\right) \le \theta_{LW} \le \arctan\left(\dfrac{0.5 \cdot a_W \cdot \cos(\theta_{WT})}{a_L \cdot \cos(\theta_{LW})}\right) \end{cases} \tag{S9}$$

$$\begin{cases} l_{10} = \dfrac{(W + a_W) \cdot \cos(\theta_{WT})}{\sin(\theta_{LW})} + (L + 0.5 \cdot a_L) \cdot \cos(\theta_{LT}) \cdot \cos(\theta_{LW}) + \\ -(W + a_W) \cdot \cos(\theta_{WT}) \cdot \cot(\theta_{LW}) \\ l_{10\_E} = (L + 0.5 \cdot a_L) \cdot \cos(\theta_{LT}) \cdot \cos(\theta_{LW}) + (W + a_W) \cdot \cos(\theta_{WT}) \cdot \sin(\theta_{LW}) \\ \arctan\left(\dfrac{W \cdot \cos(\theta_{WT})}{0.5 \cdot a_L \cdot \cos(\theta_{LW})}\right) \le \theta_{LW} \le \arctan\left(\dfrac{(W + a_W) \cdot \cos(\theta_{WT})}{0.5 \cdot a_L \cdot \cos(\theta_{LW})}\right) \end{cases} \tag{S10}$$

$$\begin{cases} l_{11} = \dfrac{a_W \cdot \cos(\theta_{WT})}{\sin(\theta_{LW})} + L \cdot \cos(\theta_{LT}) \cdot \cos(\theta_{LW}) - a_W \cdot \cos(\theta_{WT}) \cdot \cot(\theta_{LW}) + \\ +W \cdot \cos(\theta_{WT}) \cdot \cos(\theta_{LW}) \\ l_{11\_E} = (L + a_L) \cdot \cos(\theta_{LT}) \cdot \cos(\theta_{LW}) + (W + a_W) \cdot \cos(\theta_{WT}) \cdot \sin(\theta_{LW}) \\ \arctan\left(\dfrac{a_W \cdot \cos(\theta_{WT})}{L \cdot \cos(\theta_{LW})}\right) \le \theta_{LW} \le \dfrac{\pi}{2} \end{cases} \tag{S11}$$

$$\begin{cases} l_{12} = \boldsymbol{L}\cdot\cos(\boldsymbol{\theta_{LT}})\cdot\cos(\boldsymbol{\theta_{LW}}) + \dfrac{\boldsymbol{a_L}\cdot\cos(\boldsymbol{\theta_{LT}})}{\cos(\boldsymbol{\theta_{LW}})} + \boldsymbol{a_W}\cdot\cos(\boldsymbol{\theta_{WT}})\cdot\cos(\boldsymbol{\theta_{LW}}) \\ l_{12\_E} = (\boldsymbol{L}+\boldsymbol{a_L})\cdot\cos(\boldsymbol{\theta_{LT}})\cdot\cos(\boldsymbol{\theta_{LW}}) + (\boldsymbol{a_W})\cdot\cos(\boldsymbol{\theta_{WT}})\cdot\sin(\boldsymbol{\theta_{LW}}) \\ 0 \le \boldsymbol{\theta_{LW}} \le \arctan\left(\dfrac{\boldsymbol{a_W}\cdot\cos(\boldsymbol{\theta_{WT}})}{\boldsymbol{L}\cdot\cos(\boldsymbol{\theta_{LW}})}\right) \end{cases} \tag{S12}$$

Concentration gradient parallel to the width direction (W) of the CS_T

Equations that describe streamlines in the LW plane for a flow parallel to the width direction

$$\begin{cases} l_{13} = \boldsymbol{W}\cdot\cos(\boldsymbol{\theta_{WT}})\cdot\cos(\boldsymbol{\theta_{LW}}) + \dfrac{\boldsymbol{a_W}\cdot\cos(\boldsymbol{\theta_{WT}})}{\sin(\boldsymbol{\theta_{LW}})} + \\ +\boldsymbol{L}\cdot\cos(\boldsymbol{\theta_{LT}})\cdot\cos(\boldsymbol{\theta_{LW}}) - \boldsymbol{a_W}\cdot\cos(\boldsymbol{\theta_{WT}})\cdot\cot(\boldsymbol{\theta_{LW}}) + \boldsymbol{a_W}\cdot\cos(\boldsymbol{\theta_{WT}})\cdot\cos(\boldsymbol{\theta_{LW}}) \\ l_{13\_E} = (\boldsymbol{L})\cdot\cos(\boldsymbol{\theta_{LT}})\cdot\sin(\boldsymbol{\theta_{LW}}) + (\boldsymbol{W}+2\cdot\boldsymbol{a_W})\cdot\cos(\boldsymbol{\theta_{WT}})\cdot\cos(\boldsymbol{\theta_{LW}}) \\ \arctan\left(\dfrac{\boldsymbol{a_W}\cdot\cos(\boldsymbol{\theta_{WT}})}{\boldsymbol{L}\cdot\cos(\boldsymbol{\theta_{LW}})}\right) \le \boldsymbol{\theta_{LW}} \le \dfrac{\boldsymbol{\pi}}{2} \end{cases} \tag{S13}$$

$$\begin{cases} l_{14} = \dfrac{\boldsymbol{a_L}\cdot\cos(\boldsymbol{\theta_{LT}})}{\cos(\boldsymbol{\theta_{LW}})} + \boldsymbol{W}\cdot\cos(\boldsymbol{\theta_{WT}})\cdot\cos(\boldsymbol{\theta_{LW}}) - \boldsymbol{a_L}\cdot\cos(\boldsymbol{\theta_{LT}})\cdot\tan(\boldsymbol{\theta_{LW}}) + \boldsymbol{a_W}\cdot\cos(\boldsymbol{\theta_{WT}})\cdot\cos(\boldsymbol{\theta_{LW}}) \\ l_{14\_E} = (\boldsymbol{a_L})\cdot\cos(\boldsymbol{\theta_{LT}})\cdot\sin(\boldsymbol{\theta_{LW}}) + (\boldsymbol{W}+\boldsymbol{a_W})\cdot\cos(\boldsymbol{\theta_{WT}})\cdot\cos(\boldsymbol{\theta_{LW}}) \\ 0 \le \boldsymbol{\theta_{LW}} \le \arctan\left(\dfrac{\boldsymbol{W}\cdot\cos(\boldsymbol{\theta_{WT}})}{\boldsymbol{a_L}\cdot\cos(\boldsymbol{\theta_{LW}})}\right) \end{cases} \tag{S14}$$

$$\begin{cases} l_{15} = \dfrac{\boldsymbol{a_L}\cdot\cos(\boldsymbol{\theta_{LT}})}{\cos(\boldsymbol{\theta_{LW}})} + (\boldsymbol{W}+\boldsymbol{a_W})\cdot\cos(\boldsymbol{\theta_{WT}})\cdot\cos(\boldsymbol{\theta_{LW}}) - \boldsymbol{a_L}\cdot\cos(\boldsymbol{\theta_{LT}})\cdot\tan(\boldsymbol{\theta_{LW}}) + \boldsymbol{L}\cdot\cos(\boldsymbol{\theta_{LT}})\cdot\cos(\boldsymbol{\theta_{LW}}) \\ l_{15\_E} = (\boldsymbol{L}+\boldsymbol{a_L})\cdot\cos(\boldsymbol{\theta_{LT}})\cdot\sin(\boldsymbol{\theta_{LW}}) + (\boldsymbol{W}+\boldsymbol{a_W})\cdot\cos(\boldsymbol{\theta_{WT}})\cdot\cos(\boldsymbol{\theta_{LW}}) \\ \arctan\left(\dfrac{\boldsymbol{a_W}\cdot\cos(\boldsymbol{\theta_{WT}})}{\boldsymbol{a_L}\cdot\cos(\boldsymbol{\theta_{LW}})}\right) \le \boldsymbol{\theta_{LW}} \le \arctan\left(\dfrac{(\boldsymbol{W}+2\cdot\boldsymbol{a_W})\cdot\cos(\boldsymbol{\theta_{WT}})}{\boldsymbol{a_L}\cdot\cos(\boldsymbol{\theta_{LW}})}\right) \end{cases} \tag{S15}$$

$$\begin{cases} l_{16} = \dfrac{0.5\cdot\boldsymbol{a_L}\cdot\cos(\boldsymbol{\theta_{LT}})}{\cos(\boldsymbol{\theta_{LW}})} + (\boldsymbol{W}+\boldsymbol{a_W})\cdot\cos(\boldsymbol{\theta_{WT}})\cdot\cos(\boldsymbol{\theta_{LW}}) - 0.5\cdot\boldsymbol{a_L}\cdot\cos(\boldsymbol{\theta_{LT}})\cdot\tan(\boldsymbol{\theta_{LW}}) + \\ +\boldsymbol{a_W}\cdot\cos(\boldsymbol{\theta_{WT}})\cdot\cos(\boldsymbol{\theta_{LW}}) \\ l_{16\_E} = (0.5\cdot\boldsymbol{a_L})\cdot\cos(\boldsymbol{\theta_{LT}})\cdot\sin(\boldsymbol{\theta_{LW}}) + (\boldsymbol{W}+2\cdot\boldsymbol{a_W})\cdot\cos(\boldsymbol{\theta_{WT}})\cdot\cos(\boldsymbol{\theta_{LW}}) \\ \arctan\left(\dfrac{\boldsymbol{a_W}\cdot\cos(\boldsymbol{\theta_{WT}})}{0.5\cdot\boldsymbol{a_L}\cdot\cos(\boldsymbol{\theta_{LW}})}\right) \le \boldsymbol{\theta_{LW}} \le \arctan\left(\dfrac{(\boldsymbol{W}+2\cdot\boldsymbol{a_W})\cdot\cos(\boldsymbol{\theta_{WT}})}{0.5\cdot\boldsymbol{a_L}\cdot\cos(\boldsymbol{\theta_{LW}})}\right) \end{cases} \tag{S16}$$

$$\begin{cases} l_{17} = \dfrac{(W+a_W)\cdot\cos(\theta_{WT})}{\sin(\theta_{LW})} + (L+a_L)\cdot\cos(\theta_{LT})\cdot\cos(\theta_{LW}) - (W+a_W)\cdot\cos(\theta_{WT})\cdot\cot(\theta_{LW}) + \\ +0.5\cdot a_W\cdot\cos(\theta_{WT})\cdot\cos(\theta_{LW}) \\ l_{17\_E} = (L+a_L)\cdot\cos(\theta_{LT})\cdot\sin(\theta_{LW}) + (W+0.5\cdot a_W)\cdot\cos(\theta_{WT})\cdot\cos(\theta_{LW}) \\ \arctan\left(\dfrac{W\cdot\cos(\theta_{WT})}{a_L\cdot\cos(\theta_{LW})}\right) \le \theta_{LW} \le \arctan\left(\dfrac{(W+a_W)\cdot\cos(\theta_{WT})}{a_L\cdot\cos(\theta_{LW})}\right) \end{cases} \tag{S17}$$

$$\begin{cases} l_{18} = \dfrac{a_W\cdot\cos(\theta_{WT})}{\sin(\theta_{LW})} + (L+a_L)\cdot\cos(\theta_{LT})\cdot\cos(\theta_{LW}) - 0.5\cdot a_W\cdot\cos(\theta_{WT})\cdot\cot(\theta_{LW}) + \\ +0.5\cdot a_W\cdot\cos(\theta_{WT})\cdot\cos(\theta_{LW}) \\ l_{18\_E} = (L+a_L)\cdot\cos(\theta_{LT})\cdot\sin(\theta_{LW}) + (0.5\cdot a_W)\cdot\cos(\theta_{WT})\cdot\cos(\theta_{LW}) \\ \arctan\left(\dfrac{a_W\cdot\cos(\theta_{WT})}{(L+a_L)\cdot\cos(\theta_{LW})}\right) \le \theta_{LW} \le \arctan\left(\dfrac{a_W\cdot\cos(\theta_{WT})}{a_L\cdot\cos(\theta_{LW})}\right) \end{cases} \tag{S18}$$

Equations that describe the streamlines in the WT plane for a flow parallel to the width direction

$$\begin{cases} l_{19} = T\cdot\cos(\theta_{LT})\cdot\cos(\theta_{WT}) + \dfrac{a_W\cdot\cos(\theta_{LW})}{\cos(\theta_{WT})} + a_W\cdot\cos(\theta_{LW})\cdot\tan(\theta_{WT}) + \dfrac{a_T\cdot\cos(\theta_{LT})}{\sin(\theta_{WT})} + \\ +W\cdot\cos(\theta_{LW})\cdot\cos(\theta_{WT}) - a_T\cdot\cos(\theta_{LT})\cdot\cot(\theta_{WT}) + T\cdot\cos(\theta_{LT})\cdot\cos(\theta_{WT}) \\ l_{19\_E} = (T+a_T)\cdot\cos(\theta_{LT})\cdot\sin(\theta_{LW}) + (W+a_W)\cdot\cos(\theta_{WT})\cdot\cos(\theta_{LW}) \\ \arctan\left(\dfrac{(T+a_T)\cdot\cos(\theta_{LT})}{a_W\cdot\cos(\theta_{LW})}\right) \le \theta_{WT} \le \arctan\left(\dfrac{(1.5\cdot T+a_T)\cdot\cos(\theta_{LT})}{a_W\cdot\cos(\theta_{LW})}\right) \end{cases} \tag{S19}$$

$$\begin{cases} l_{20} = T\cdot\cos(\theta_{LT})\cdot\cos(\theta_{WT}) + \dfrac{a_W\cdot\cos(\theta_{LW})}{\cos(\theta_{WT})} + \\ +(3\cdot T+2\cdot a_T)\cdot\cos(\theta_{LT})\cdot\cos(\theta_{WT}) - a_W\cdot\cos(\theta_{LW})\cdot\tan(\theta_{WT}) + \dfrac{a_T\cdot\cos(\theta_{LT})}{\sin(\theta_{WT})} + \\ +W\cdot\cos(\theta_{LW})\cdot\cos(\theta_{WT}) - a_T\cdot\cos(\theta_{LT})\cdot\cot(\theta_{WT}) + (2\cdot T+a_T)\cdot\cos(\theta_{LT})\cdot\cos(\theta_{WT}) \\ l_{20\_E} = 2\cdot(T+a_T)\cdot\cos(\theta_{LT})\cdot\sin(\theta_{LW}) + (W+a_W)\cdot\cos(\theta_{WT})\cdot\cos(\theta_{LW}) \\ \arctan\left(\dfrac{2\cdot(T+a_T)\cdot\cos(\theta_{LT})}{a_W\cdot\cos(\theta_{LW})}\right) \le \theta_{WT} \le \arctan\left(\dfrac{(3\cdot T+2\cdot a_T)\cdot\cos(\theta_{LT})}{a_W\cdot\cos(\theta_{LW})}\right) \end{cases} \tag{S20}$$

$$\begin{cases} l_{21} = T\cdot\cos(\theta_{LT})\cdot\cos(\theta_{WT}) + 2\cdot\left(\dfrac{a_T\cdot\cos(\theta_{LT})}{\sin(\theta_{WT})} + a_T\cdot\cos(\theta_{LT})\cdot\cot(\theta_{WT})\right) + \\ +(2\cdot T)\cdot\cos(\theta_{LT})\cdot\cos(\theta_{WT}) + \dfrac{a_T\cdot\cos(\theta_{LT})}{\sin(\theta_{WT})} + W\cdot\cos(\theta_{LW})\cdot\cos(\theta_{WT}) + \\ -a_T\cdot\cos(\theta_{LT})\cdot\cot(\theta_{WT}) + (T+a_T)\cdot\cos(\theta_{LT})\cdot\cos(\theta_{WT}) \\ l_{21\_E} = 2\cdot(T+a_T)\cdot\cos(\theta_{LT})\cdot\sin(\theta_{LW}) + W\cdot\cos(\theta_{WT})\cdot\cos(\theta_{LW}) \\ \arctan\left(\dfrac{a_T\cdot\cos(\theta_{LT})}{W\cdot\cos(\theta_{LW})}\right) \le \theta_{WT} \le \dfrac{\pi}{2} \end{cases} \tag{S21}$$

$$\begin{cases} l_{22} = T\cdot\cos(\theta_{LT})\cdot\cos(\theta_{WT}) + 2\cdot\left(\dfrac{a_T\cdot\cos(\theta_{LT})}{\sin(\theta_{WT})} + a_T\cdot\cos(\theta_{LT})\cdot\cot(\theta_{WT})\right) + \\ +(2\cdot T)\cdot\cos(\theta_{LT})\cdot\cos(\theta_{WT}) + \dfrac{a_T\cdot\cos(\theta_{LT})}{\sin(\theta_{WT})} + W\cdot\cos(\theta_{LW})\cdot\cos(\theta_{WT}) + \\ -a_T\cdot\cos(\theta_{LT})\cdot\cot(\theta_{WT}) + 2\cdot(T+a_T)\cdot\cos(\theta_{LT})\cdot\cos(\theta_{WT}) \\ l_{22\_E} = (T+a_T)\cdot\cos(\theta_{LT})\cdot\sin(\theta_{LW}) + W\cdot\cos(\theta_{WT})\cdot\cos(\theta_{LW}) \\ \arctan\left(\dfrac{a_T\cdot\cos(\theta_{LT})}{W\cdot\cos(\theta_{LW})}\right) \le \theta_{WT} \le \dfrac{\pi}{2} \end{cases} \tag{S22}$$

$$\begin{cases} l_{23} = T\cdot\cos(\theta_{LT})\cdot\cos(\theta_{WT}) + 2\cdot\left(\dfrac{a_T\cdot\cos(\theta_{LT})}{\sin(\theta_{WT})} + a_T\cdot\cos(\theta_{LT})\cdot\cot(\theta_{WT})\right) + (2\cdot T)\cdot\cos(\theta_{LT})\cdot\cos(\theta_{WT}) + \\ +\dfrac{a_T\cdot\cos(\theta_{LT})}{\sin(\theta_{WT})} + W\cdot\cos(\theta_{LW})\cdot\cos(\theta_{WT}) - a_T\cdot\cos(\theta_{LT})\cdot\cot(\theta_{WT}) + T\cdot\cos(\theta_{LT})\cdot\cos(\theta_{WT}) \\ l_{23\_E} = (4\cdot T + 3\cdot a_T)\cdot\cos(\theta_{LT})\cdot\sin(\theta_{LW}) + W\cdot\cos(\theta_{WT})\cdot\cos(\theta_{LW}) \\ \arctan\left(\dfrac{a_T\cdot\cos(\theta_{LT})}{W\cdot\cos(\theta_{LW})}\right) \le \theta_{WT} \le \dfrac{\pi}{2} \end{cases} \tag{S23}$$

$$\begin{cases} l_{24} = T\cdot\cos(\theta_{LT})\cdot\cos(\theta_{WT}) + 2\cdot\left(\dfrac{a_T\cdot\cos(\theta_{LT})}{\sin(\theta_{WT})} + a_T\cdot\cos(\theta_{LT})\cdot\cot(\theta_{WT})\right) + \\ +(2\cdot T)\cdot\cos(\theta_{LT})\cdot\cos(\theta_{WT}) + \dfrac{a_T\cdot\cos(\theta_{LT})}{\sin(\theta_{WT})} + W\cdot\cos(\theta_{LW})\cdot\cos(\theta_{WT}) + \\ -a_T\cdot\cos(\theta_{LT})\cdot\cot(\theta_{WT}) + T\cdot\cos(\theta_{LT})\cdot\cos(\theta_{WT}) \\ l_{24\_E} = 2\cdot(T+a_T)\cdot\cos(\theta_{LT})\cdot\sin(\theta_{LW}) + W\cdot\cos(\theta_{WT})\cdot\cos(\theta_{LW}) \\ \arctan\left(\dfrac{a_T\cdot\cos(\theta_{LT})}{W\cdot\cos(\theta_{LW})}\right) \le \theta_{WT} \le \dfrac{\pi}{2} \end{cases} \tag{S24}$$

Concentration gradient parallel to the thickness direction (T) of the CS_T

Equations that describe the streamlines in the LT plane for a flow parallel to the thickness direction

$$\begin{cases} l_{25} = \dfrac{a_T\cdot\cos(\theta_{WT})}{\cos(\theta_{LT})} + 2\cdot\left(0.5\cdot(L-a_L)\cdot\cos(\theta_{LW})\cdot\cos(\theta_{LT})\right) - 2\cdot a_T\cdot\cos(\theta_{WT})\cdot\tan(\theta_{LT}) + \\ +2\cdot\dfrac{a_L\cdot\cos(\theta_{LW})}{\cos(\theta_{LT})} + 2\cdot T\cdot\cos(\theta_{WT})\cdot\cos(\theta_{LT}) - 2\cdot a_L\cdot\cos(\theta_{LW})\cdot\tan(\theta_{LT}) + 2\cdot T\cdot\cos(\theta_{WT})\cdot\cos(\theta_{LT}) \\ l_{25\_E} = (L+a_L)\cdot\cos(\theta_{LW})\cdot\sin(\theta_{LT}) + (3\cdot T + 2\cdot a_T)\cdot\cos(\theta_{WT})\cdot\cos(\theta_{LT}) \\ \arctan\left(\dfrac{T\cdot\cos(\theta_{WT})}{0.5\cdot(L-a_L)\cdot\cos(\theta_{LW})}\right) \le \theta_{LT} \le \dfrac{\pi}{2} \end{cases} \tag{S25}$$

$$\begin{cases} \boldsymbol{l}_{26} = \dfrac{(\boldsymbol{T}+\boldsymbol{a_T})\cdot\cos(\boldsymbol{\theta_{WT}})}{\cos(\boldsymbol{\theta_{LT}})} + 0.5\cdot(\boldsymbol{L}+\boldsymbol{a_L})\cdot\cos(\boldsymbol{\theta_{LW}})\cdot\cos(\boldsymbol{\theta_{LT}}) - (\boldsymbol{T}+\boldsymbol{a_T})\cdot\cos(\boldsymbol{\theta_{WT}})\cdot\tan(\boldsymbol{\theta_{LT}}) + \\ + \dfrac{(\boldsymbol{a_T})\cdot\cos(\boldsymbol{\theta_{WT}})}{\cos(\boldsymbol{\theta_{LT}})} + 0.5\cdot(\boldsymbol{L}-\boldsymbol{a_L})\cdot\cos(\boldsymbol{\theta_{LW}})\cdot\cos(\boldsymbol{\theta_{LT}}) - \boldsymbol{a_T}\cdot\cos(\boldsymbol{\theta_{WT}})\cdot\tan(\boldsymbol{\theta_{LT}}) + \\ + \boldsymbol{T}\cdot\cos(\boldsymbol{\theta_{WT}})\cdot\cos(\boldsymbol{\theta_{LT}}) + \boldsymbol{a_L}\cdot\cos(\boldsymbol{\theta_{LW}})\cdot\cos(\boldsymbol{\theta_{LT}}) + \boldsymbol{T}\cdot\cos(\boldsymbol{\theta_{WT}})\cdot\cos(\boldsymbol{\theta_{LT}}) \\ \boldsymbol{l}_{26\_\boldsymbol{E}} = (\boldsymbol{L}+\boldsymbol{a_L})\cdot\cos(\boldsymbol{\theta_{LW}})\cdot\sin(\boldsymbol{\theta_{LT}}) + (3\cdot\boldsymbol{T}+2\cdot\boldsymbol{a_T})\cdot\cos(\boldsymbol{\theta_{WT}})\cdot\cos(\boldsymbol{\theta_{LT}}) \\ \arctan\left(\dfrac{\boldsymbol{T}\cdot\cos(\boldsymbol{\theta_{WT}})}{0.5\cdot(\boldsymbol{L}-\boldsymbol{a_L})\cdot\cos(\boldsymbol{\theta_{LW}})}\right) \le \boldsymbol{\theta_{LT}} \le \dfrac{\pi}{2} \end{cases} \quad \text{(S26)}$$

$$\begin{cases} \boldsymbol{l}_{27} = (\boldsymbol{L}+\boldsymbol{a_L})\cdot\cos(\boldsymbol{\theta_{LW}})\cdot\cos(\boldsymbol{\theta_{LT}}) + \boldsymbol{T}\cdot\cos(\boldsymbol{\theta_{WT}})\cdot\cos(\boldsymbol{\theta_{LT}}) + \dfrac{\boldsymbol{a_T}\cdot\cos(\boldsymbol{\theta_{WT}})}{\cos(\boldsymbol{\theta_{LT}})} + \\ +2\cdot\left(0.5\cdot(\boldsymbol{L}-\boldsymbol{a_L})\cdot\cos(\boldsymbol{\theta_{LW}})\cdot\cos(\boldsymbol{\theta_{LT}})\right) - 2\cdot\boldsymbol{a_T}\cdot\cos(\boldsymbol{\theta_{WT}})\cdot\tan(\boldsymbol{\theta_{LT}}) + \\ +2\cdot\dfrac{\boldsymbol{a_L}\cdot\cos(\boldsymbol{\theta_{LW}})}{\cos(\boldsymbol{\theta_{LT}})} + 2\cdot\boldsymbol{T}\cdot\cos(\boldsymbol{\theta_{WT}})\cdot\cos(\boldsymbol{\theta_{LT}}) - 2\cdot\boldsymbol{a_L}\cdot\cos(\boldsymbol{\theta_{LW}})\cdot\tan(\boldsymbol{\theta_{LT}}) + \\ +2\cdot\boldsymbol{T}\cdot\cos(\boldsymbol{\theta_{WT}})\cdot\cos(\boldsymbol{\theta_{LT}}) \\ \boldsymbol{l}_{27\_\boldsymbol{E}} = (\boldsymbol{L}+\boldsymbol{a_L})\cdot\cos(\boldsymbol{\theta_{LW}})\cdot\sin(\boldsymbol{\theta_{LT}}) + (3\cdot\boldsymbol{T}+2\cdot\boldsymbol{a_T})\cdot\cos(\boldsymbol{\theta_{WT}})\cdot\cos(\boldsymbol{\theta_{LT}}) \\ \arctan\left(\dfrac{\boldsymbol{T}\cdot\cos(\boldsymbol{\theta_{WT}})}{0.5\cdot(\boldsymbol{L}-\boldsymbol{a_L})\cdot\cos(\boldsymbol{\theta_{LW}})}\right) \le \boldsymbol{\theta_{LT}} \le \arctan\left(\dfrac{\boldsymbol{T}\cdot\cos(\boldsymbol{\theta_{WT}})}{0.5\cdot(\boldsymbol{L}+\boldsymbol{a_L})\cdot\cos(\boldsymbol{\theta_{LW}})}\right) \end{cases} \quad \text{(S27)}$$

$$\begin{cases} \boldsymbol{l}_{28} = \boldsymbol{T}\cdot\cos(\boldsymbol{\theta_{WT}})\cdot\cos(\boldsymbol{\theta_{LT}}) + \\ +2\cdot\left(\dfrac{\boldsymbol{a_T}\cdot\cos(\boldsymbol{\theta_{WT}})}{\sin(\boldsymbol{\theta_{LT}})} + 0.5\cdot(\boldsymbol{L}+\boldsymbol{a_L})\cdot\cos(\boldsymbol{\theta_{LW}})\cdot\cos(\boldsymbol{\theta_{LT}}) - \boldsymbol{a_T}\cdot\cos(\boldsymbol{\theta_{WT}})\cdot\cot(\boldsymbol{\theta_{LT}})\right) + \\ + \dfrac{\boldsymbol{a_L}\cdot\cos(\boldsymbol{\theta_{LW}})}{\cos(\boldsymbol{\theta_{LT}})} + \boldsymbol{T}\cdot\cos(\boldsymbol{\theta_{WT}})\cdot\cos(\boldsymbol{\theta_{LT}}) - \boldsymbol{a_L}\cdot\cos(\boldsymbol{\theta_{LW}})\cdot\tan(\boldsymbol{\theta_{LT}}) + \\ + \boldsymbol{a_L}\cdot\cos(\boldsymbol{\theta_{LW}})\cdot\cos(\boldsymbol{\theta_{LT}}) - \boldsymbol{a_T}\cdot\cos(\boldsymbol{\theta_{WT}})\cdot\cos(\boldsymbol{\theta_{LT}}) + \boldsymbol{T}\cdot\cos(\boldsymbol{\theta_{WT}})\cdot\cos(\boldsymbol{\theta_{LT}}) \\ \boldsymbol{l}_{28\_\boldsymbol{E}} = (\boldsymbol{L}+2\cdot\boldsymbol{a_L})\cdot\cos(\boldsymbol{\theta_{LW}})\cdot\sin(\boldsymbol{\theta_{LT}}) + (3\cdot\boldsymbol{T}+2\cdot\boldsymbol{a_T})\cdot\cos(\boldsymbol{\theta_{WT}})\cdot\cos(\boldsymbol{\theta_{LT}}) \\ \arctan\left(\dfrac{\boldsymbol{T}\cdot\cos(\boldsymbol{\theta_{WT}})}{0.5\cdot(\boldsymbol{L}-\boldsymbol{a_L})\cdot\cos(\boldsymbol{\theta_{LW}})}\right) \le \boldsymbol{\theta_{LT}} \le \dfrac{\pi}{2} \end{cases} \quad \text{(S28)}$$

$$\begin{cases} \boldsymbol{l}_{29} = \boldsymbol{L}\cdot\cos(\boldsymbol{\theta_{LW}})\cdot\cos(\boldsymbol{\theta_{LT}}) + \boldsymbol{T}\cdot\cos(\boldsymbol{\theta_{WT}})\cdot\cos(\boldsymbol{\theta_{LT}}) + \dfrac{\boldsymbol{a_T}\cdot\cos(\boldsymbol{\theta_{WT}})}{\cos(\boldsymbol{\theta_{LT}})} + 0.5\cdot(\boldsymbol{L}-\boldsymbol{a_L})\cdot\cos(\boldsymbol{\theta_{LW}})\cdot\cos(\boldsymbol{\theta_{LT}}) + \\ -\boldsymbol{a_T}\cdot\cos(\boldsymbol{\theta_{WT}})\cdot\tan(\boldsymbol{\theta_{LT}}) + \dfrac{\boldsymbol{a_L}\cdot\cos(\boldsymbol{\theta_{LW}})}{\cos(\boldsymbol{\theta_{LT}})} + \boldsymbol{T}\cdot\cos(\boldsymbol{\theta_{WT}})\cdot\cos(\boldsymbol{\theta_{LT}}) - \boldsymbol{a_L}\cdot\cos(\boldsymbol{\theta_{LW}})\cdot\tan(\boldsymbol{\theta_{LT}}) + \\ + \boldsymbol{a_L}\cdot\cos(\boldsymbol{\theta_{LW}})\cdot\cos(\boldsymbol{\theta_{LT}}) + \dfrac{\boldsymbol{a_T}\cdot\cos(\boldsymbol{\theta_{WT}})}{\cos(\boldsymbol{\theta_{LT}})} + 0.5\cdot(\boldsymbol{L}-\boldsymbol{a_L})\cdot\cos(\boldsymbol{\theta_{LW}})\cdot\cos(\boldsymbol{\theta_{LT}}) + \\ -\boldsymbol{a_T}\cdot\cos(\boldsymbol{\theta_{WT}})\cdot\tan(\boldsymbol{\theta_{LT}}) + \boldsymbol{T}\cdot\cos(\boldsymbol{\theta_{WT}})\cdot\cos(\boldsymbol{\theta_{LT}}) \\ \boldsymbol{l}_{29\_\boldsymbol{E}} = \boldsymbol{L}\cdot\cos(\boldsymbol{\theta_{LW}})\cdot\sin(\boldsymbol{\theta_{LT}}) + (3\cdot\boldsymbol{T}+2\cdot\boldsymbol{a_T})\cdot\cos(\boldsymbol{\theta_{WT}})\cdot\cos(\boldsymbol{\theta_{LT}}) \\ \arctan\left(\dfrac{\boldsymbol{T}\cdot\cos(\boldsymbol{\theta_{WT}})}{0.5\cdot(\boldsymbol{L}-\boldsymbol{a_L})\cdot\cos(\boldsymbol{\theta_{LW}})}\right) \le \boldsymbol{\theta_{LT}} \le \dfrac{\pi}{2} \end{cases} \quad \text{(S29)}$$

$$\begin{cases} l_{30} = (T + a_T)\cdot\cos(\theta_{WT})\cdot\cos(\theta_{LT}) + L\cdot\cos(\theta_{LW})\cdot\cos(\theta_{LT}) \\ l_{30\_E} = (L + a_L)\cdot\cos(\theta_{LW})\cdot\sin(\theta_{LT}) + (T + a_T)\cdot\cos(\theta_{WT})\cdot\cos(\theta_{LT}) \\ 0 \le \theta_{LT} \le \arctan\left(\dfrac{T\cdot\cos(\theta_{WT})}{a_L\cdot\cos(\theta_{LW})}\right) \end{cases} \tag{S30}$$

Equations that describe the streamlines in the WT plane for a flow parallel to the thickness direction

$$\begin{cases} l_{31} = T\cdot\cos(\theta_{LT})\cdot\cos(\theta_{WT}) + \dfrac{a_T\cdot\cos(\theta_{LT})}{\cos(\theta_{WT})} + W\cdot\cos(\theta_{LW})\cdot\cos(\theta_{WT}) - a_T\cdot\cos(\theta_{LT})\cdot\tan(\theta_{WT}) + \\ +T\cdot\cos(\theta_{LT})\cdot\cos(\theta_{WT}) + W\cdot\cos(\theta_{LW})\cdot\cos(\theta_{WT}) \\ l_{31\_E} = (2\cdot T + a_T)\cdot\cos(\theta_{LT})\cdot\cos(\theta_{WT}) \\ \arctan\left(\dfrac{a_T\cdot\cos(\theta_{LT})}{W\cdot\cos(\theta_{LW})}\right) \le \theta_{WT} \le \dfrac{\pi}{2} \end{cases} \tag{S31}$$

$$\begin{cases} l_{32} = W\cdot\cos(\theta_{LW})\cdot\cos(\theta_{WT}) + T\cdot\cos(\theta_{LT})\cdot\cos(\theta_{WT}) + \dfrac{a_T\cdot\cos(\theta_{LT})}{\cos(\theta_{WT})} + \\ +W\cdot\cos(\theta_{LW})\cdot\cos(\theta_{WT}) - a_T\cdot\cos(\theta_{LT})\cdot\tan(\theta_{WT}) + T\cdot\cos(\theta_{LT})\cdot\cos(\theta_{WT}) + W\cdot\cos(\theta_{LW})\cdot\cos(\theta_{WT}) \\ l_{32\_E} = (2\cdot T + a_T)\cdot\cos(\theta_{LT})\cdot\cos(\theta_{WT}) + W\cdot\cos(\theta_{LW})\cdot\cos(\theta_{WT}) \\ \arctan\left(\dfrac{a_T\cdot\cos(\theta_{LT})}{W\cdot\cos(\theta_{LW})}\right) \le \theta_{WT} \le \dfrac{\pi}{2} \end{cases} \tag{S32}$$

$$\begin{cases} l_{33} = W\cdot\cos(\theta_{LW})\cdot\cos(\theta_{WT}) + (T + a_T)\cdot\cos(\theta_{LT})\cdot\cos(\theta_{WT}) + \\ +W\cdot\cos(\theta_{LW})\cdot\cos(\theta_{WT}) + (T + a_T)\cdot\cos(\theta_{LT})\cdot\cos(\theta_{WT}) \\ l_{33\_E} = 2\cdot(T + a_T)\cdot\cos(\theta_{LT})\cdot\cos(\theta_{WT}) \\ 0 \le \theta_{WT} \le \arctan\left(\dfrac{a_T\cdot\cos(\theta_{LT})}{W\cdot\cos(\theta_{LW})}\right) \end{cases} \tag{S33}$$

$$\begin{cases} l_{34} = \dfrac{a_T\cdot\cos(\theta_{LT})}{\cos(\theta_{WT})} + W\cdot\cos(\theta_{LW})\cdot\cos(\theta_{WT}) - a_T\cdot\cos(\theta_{LT})\cdot\tan(\theta_{WT}) + \\ +(2\cdot T + a_T)\cdot\cos(\theta_{LT})\cdot\cos(\theta_{WT}) + W\cdot\cos(\theta_{LW})\cdot\cos(\theta_{WT}) \\ l_{34\_E} = 2\cdot(T + a_T)\cdot\cos(\theta_{LT})\cdot\cos(\theta_{WT}) \\ \arctan\left(\dfrac{a_T\cdot\cos(\theta_{LT})}{W\cdot\cos(\theta_{LW})}\right) \le \theta_{WT} \le \dfrac{\pi}{2} \end{cases} \tag{S34}$$

$$\begin{cases} l_{35} = T\cdot\cos(\theta_{LT})\cdot\cos(\theta_{WT}) + W\cdot\cos(\theta_{LW})\cdot\cos(\theta_{WT}) + (T + a_T)\cdot\cos(\theta_{LT})\cdot\cos(\theta_{WT}) + \dfrac{W\cdot\cos(\theta_{LW})}{\cos(\theta_{WT})} + \\ +(T + a_T)\cdot\cos(\theta_{LT})\cdot\cos(\theta_{WT}) + W\cdot\cos(\theta_{LW})\cdot\cos(\theta_{WT}) \\ l_{35\_E} = (3\cdot T + 2\cdot a_T)\cdot\cos(\theta_{LT})\cdot\cos(\theta_{WT}) \\ \arctan\left(\dfrac{a_T\cdot\cos(\theta_{LT})}{W\cdot\cos(\theta_{LW})}\right) \le \theta_{WT} \le \dfrac{\pi}{2} \end{cases} \tag{S35}$$

$$\begin{cases} l_{36} = T \cdot \cos(\theta_{LT}) \cdot \cos(\theta_{WT}) + \dfrac{a_T \cdot \cos(\theta_{LT})}{\cos(\theta_{WT})} + W \cdot \cos(\theta_{LW}) \cdot \cos(\theta_{WT}) + \\ -a_T \cdot \cos(\theta_{LT}) \cdot \tan(\theta_{WT}) + (T + a_T) \cdot \cos(\theta_{LT}) \cdot \cos(\theta_{WT}) + W \cdot \cos(\theta_{LW}) \cdot \cos(\theta_{WT}) + \\ +(T + a_T) \cdot \cos(\theta_{LT}) \cdot \cos(\theta_{WT}) + W \cdot \cos(\theta_{LW}) \cdot \cos(\theta_{WT}) + T \cdot \cos(\theta_{LT}) \cdot \cos(\theta_{WT}) \\ l_{36\_E} = (3 \cdot T + 2 \cdot a_T) \cdot \cos(\theta_{LT}) \cdot \cos(\theta_{WT}) \\ 0 \le \theta_{WT} \le \arctan\left(\dfrac{a_T \cdot \cos(\theta_{LT})}{W \cdot \cos(\theta_{LW})}\right) \end{cases} \tag{S36}$$

Assessment of the tortuosity in the collagen matrix

The tortuosity within the collagen matrix depends on the geometric characteristics of the ellipse that describes the collagen cross sections.

In order to calculate the length of the elliptical trajectories of the water molecule in the collagen matrix by means of Eq. (4), we determined the dimensions of the semi-minor and semi-major axes of the ellipse that describes the collagen cross sections.

Concentration gradient parallel to the longitudinal axis (L) of the CS_T

For a flow parallel to the longitudinal direction in the LT plane, the major axis of the ellipse is obtained as follows:

$$a = \frac{d}{2 \cdot \cos(\theta_{LW}) \cdot \sin(\theta_{LT})} \tag{S37}$$

while the minor axis of the ellipse is calculated as follows:

$$b = \frac{d}{2} \tag{S38}$$

For a flow parallel to the longitudinal direction in the LW plane, the major axis of the ellipse is obtained as follows:

$$a = \frac{d}{2 \cdot \sin(\theta_{LW}) \cdot \cos(\theta_{LT})} \tag{S39}$$

while the minor axis of the ellipse is calculated as follows

$$b = \frac{d}{2} \tag{S40}$$

Concentration gradient parallel to the width direction (W) of the CS_T

For a flow parallel to the width direction in the LW plane, the major and minor axes of the ellipse, respectively, are calculated as follows:

$$\boldsymbol{a} = \frac{\boldsymbol{d}}{2 \cdot \cos(\boldsymbol{\theta}_{\boldsymbol{LW}}) \cdot \cos(\boldsymbol{\theta}_{\boldsymbol{LT}})} \tag{S41}$$

$$\boldsymbol{b} = \frac{\boldsymbol{d}}{2} \tag{S42}$$

For a flow parallel to the width direction in the WT plane, the major and minor axes of the ellipse, respectively, are calculated as follows:

$$\boldsymbol{a} = \frac{\boldsymbol{d}}{2 \cdot \cos(\boldsymbol{\theta}_{\boldsymbol{LW}}) \cdot \cos(\boldsymbol{\theta}_{\boldsymbol{LT}})} \tag{S43}$$

$$\boldsymbol{b} = \frac{\boldsymbol{d}}{2} \tag{S44}$$

Concentration gradient parallel to the thickness direction (T) of the CS_T

For a flow parallel to the thickness direction in the LT plane, the major and minor axes of the ellipse, respectively, are calculated as follows:

$$\boldsymbol{a} = \frac{\boldsymbol{d}}{2 \cdot \cos(\boldsymbol{\theta}_{\boldsymbol{LW}}) \cdot \cos(\boldsymbol{\theta}_{\boldsymbol{LT}})} \tag{S45}$$

$$\boldsymbol{b} = \frac{\boldsymbol{d}}{2} \tag{S46}$$

For a flow parallel to the thickness direction in the WT plane, the major and minor axes of the ellipse, respectively, are calculated as follows:

$$\boldsymbol{a} = \frac{\boldsymbol{d}}{2 \cdot \cos(\boldsymbol{\theta}_{\boldsymbol{LW}}) \cdot \cos(\boldsymbol{\theta}_{\boldsymbol{LT}})} \tag{S47}$$

$$\boldsymbol{b} = \frac{\boldsymbol{d}}{2} \tag{S48}$$

Assessment of the constrictivity

In each plane, we calculated the variations of the passageway available for the diffusion of the water molecule between collagen fibrils due to the inclination of the collagen - apatite unit cell with respect to the trabecula axes. Below we report the relations that allow to calculate the constrictivity in each plane by means of Eq. (5).

In the WT plane the minimum and maximum cross section are assessed as follows:

$$\min \boldsymbol{cross}\ \text{sec}\boldsymbol{tion} = \frac{\boldsymbol{a_c}}{\cos(\boldsymbol{\theta_{LW}})} \tag{S49}$$

$$\max \boldsymbol{cross}\ \text{sec}\boldsymbol{tion} = \frac{\boldsymbol{a_T} - \boldsymbol{d}}{2 \cdot \cos(\boldsymbol{\theta_{LW}}) \cdot \cos(\boldsymbol{\theta_{LT}})} \tag{S50}$$

In the LT plane the minimum and maximum cross section are assessed as follows:

$$\min \boldsymbol{cross}\ \text{sec}\boldsymbol{tion} = \frac{\boldsymbol{a_c}}{\cos(\boldsymbol{\theta_{LW}})} \tag{S51}$$

$$\max \boldsymbol{cross}\ \text{sec}\boldsymbol{tion} = \frac{\boldsymbol{a_T} - \boldsymbol{d}}{2 \cdot \cos(\boldsymbol{\theta_{LW}}) \cdot \cos(\boldsymbol{\theta_{LT}})} \tag{S52}$$

In the LW plane the minimum and maximum cross section are assessed as follows:

$$\min \boldsymbol{cross}\ \text{sec}\boldsymbol{tion} = \frac{\boldsymbol{a_c}}{\cos(\boldsymbol{\theta_{LW}})} \tag{S53}$$

$$\max \boldsymbol{cross}\ \text{sec}\boldsymbol{tion} = \frac{\boldsymbol{a_W} - \boldsymbol{n} \cdot (\boldsymbol{d} + \boldsymbol{a_c})}{\cos(\boldsymbol{\theta_{LW}}) \cdot \cos(\boldsymbol{\theta_{LT}})} \tag{S54}$$

where **n** is equal to the number of collagen fibrils between the apatite platelets in the width direction.

Supplementary tables

Table S1. Range of values for the dimensions of the apatite platelets (L, W, T), distances between the mineral crystals (a_L, a_W, a_T), collagen fibril diameter (d) and distance between the collagen fibrils (a_c) implemented as input in the equation system.

**Parameters**	**Value (nm)**	**References**
L	(70-130)	[16]
W	(10-80)	[13]
T	(2.5-5)	[13]
a_L	(4-64)	[13]
a_W	(5-20)	[15]
a_T	(2-3.5)	[15]
d	(1.1-1.4)	[8]
a_c	(1-1.5)	[10]

Table S2. Average values of the apatite platelets dimensions (L, W, T), distances between the mineral crystals (a_L, a_W, a_T), collagen fibril diameter (d) and distance between the collagen fibrils (a_c) achieved in output from the equation system for each degree of Volume Fraction.

**Parameters**	V_{f_A}**= 0.07**	V_{f_A} **=0.32**	V_{f_A} **=0.42**
L (nm)	70.55	92.09	105.28
W (nm)	10.83	39.91	44.20
T (nm)	2.57	3.49	3.73
a_L (nm)	63.45	41.91	28.72
a_W (nm)	18.73	12.17	10.12
a_T (nm)	4.42	2.23	1.90
d (nm)	1.2390	1.2391	1.2391
a_c (nm)	1.16	1.18	1.19

Supplementary results

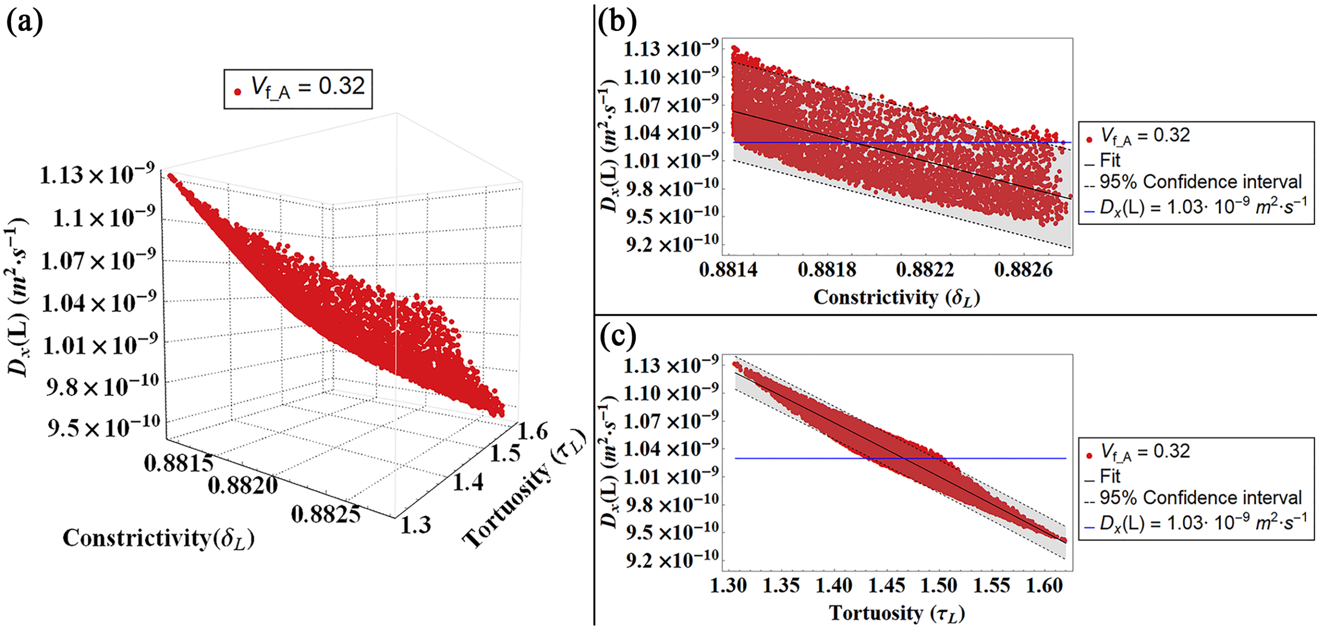

Figure S1. Diffusion coefficient for a flow parallel to the longitudinal axis (L) represented in function of the constrictivity and tortuosity factors for the intermediate mineralization degree, i.e. $V_{f\_A} = 0.32$. In Fig. (a) 3-D plot of the diffusivity D_L versus constrictivity and tortuosity, in Fig. (b) 2-D plot of D_L versus the constrictivity and in (c) 2-D plot of D_L versus the tortuosity. The colour bands in the 2-D plots indicates the Confidence Interval at 95 percent. The continuous blue line represents the Diffusion coefficient of the collagen-apatite porosity achieved in a previous study by means of a genetic algorithm [18].

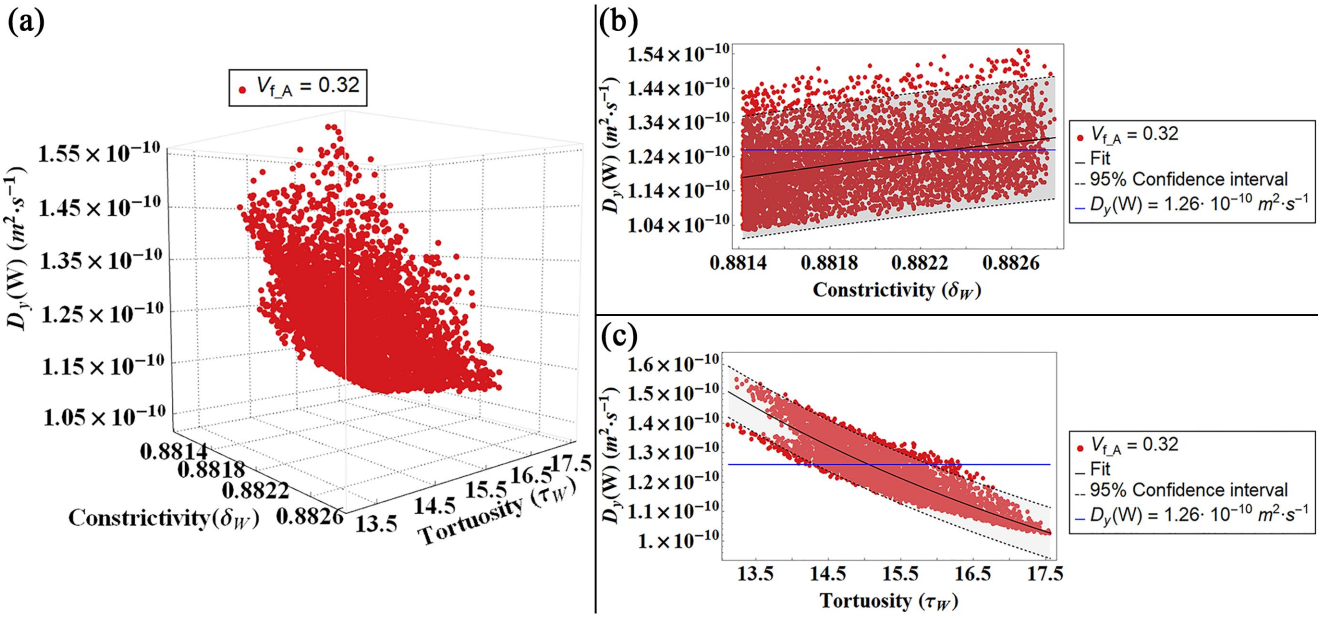

Figure S2. Diffusion coefficient for a flow parallel to the width direction (W) represented in function of the constrictivity and tortuosity factors for the intermediate mineralization degree, i.e. $V_{f\_A} = 0.32$. In Fig. (a) 3-D plot of the diffusivity D_W versus constrictivity and tortuosity, in Fig. (b) 2-D plot of D_W versus the constrictivity and in (c) 2-D plot of D_W versus the tortuosity. The colour bands in the 2-D plots indicates the Confidence Interval at 95 percent. The continuous blue line represents the Diffusion coefficient of the collagen-apatite porosity achieved in a previous study by means of a genetic algorithm [18].

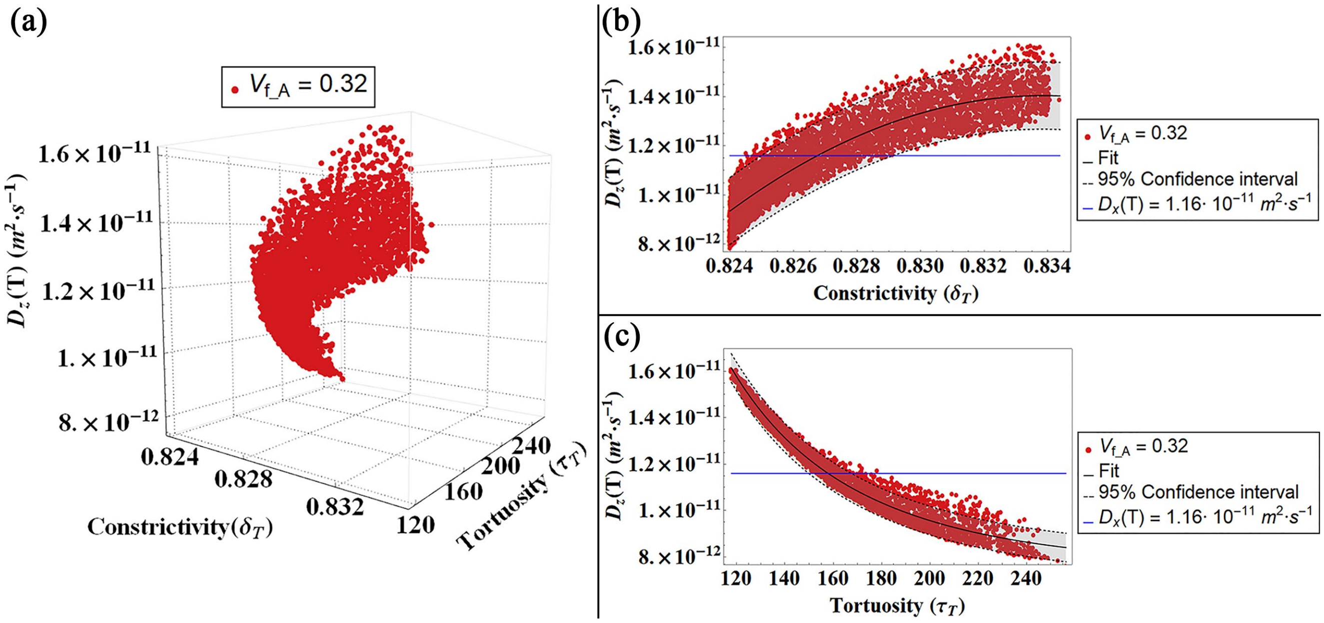

Figure S3. Diffusion coefficient for a flow parallel to the thickness direction (T) represented in function of the constrictivity and tortuosity factors for the intermediate mineralization degree, i.e. $V_{f\_A} = 0.32$. In Fig. (a) 3-D plot of the diffusivity D_T versus constrictivity and tortuosity, in Fig. (b) 2-D plot of D_T versus the constrictivity and in (c) 2-D plot of D_T versus the tortuosity. The colour bands in the 2-D plots indicates the Confidence Interval at 95 percent. The continuous blue line represents the Diffusion coefficient of the collagen-apatite porosity achieved in a previous study by means of a genetic algorithm [18].
